# The effect between metabolic syndrome and life expectancy after cancer diagnosis: Catalan cohort study

**DOI:** 10.1186/s12889-025-21437-9

**Published:** 2025-01-16

**Authors:** Tomàs López-Jiménez, Oleguer Plana-Ripoll, Talita Duarte-Salles, Anna Palomar-Cros, Diana Puente

**Affiliations:** 1 Fundació Institut Universitari per a la recerca a l’Atenció Primària de Salut Jordi Gol i Gurina (IDIAPJGol), Gran Via de Les Corts Catalanes, 587 Àtic, 08007 Barcelona, Spain; 2 Programa de Doctorat en Metodologia de la Recerca Biomèdica i Salut Pública, Universitat Autònoma de Barcelona, Barcelona (Cerdanyola del Vallès), Spain; 3 Department of Clinical Epidemiology, Aarhus University and Aarhus University Hospital, Aarhus, Denmark; 4 National Center for Register-Based Research, Aarhus University, Aarhus, Denmark; 5 Department of Medical Informatics, Erasmus University Medical Center, Rotterdam, Netherlands; 6 Universitat Autònoma de Barcelona, Bellaterra (Cerdanyola de Vallès), Barcelona, Spain

**Keywords:** Metabolic syndrome, Cancer, Remaining life expectancy, Survival

## Abstract

This study examines remaining life expectancy (RLE) after a cancer diagnosis, focusing on age, sex, cancer type, and metabolic syndrome (MS) components, using data from the SIDIAP database in Catalonia (2006–2017). RLE was analyzed for 13 cancer types, stratified by sex and MS components. The cohort study includes 183,364 individuals followed from diagnosis until death, transfer, or study end (December 2017). RLE at age 68 (median diagnosis age) was calculated based on MS components (0, 1, 2, and ≥ 3). Men aged 68 with 0 MS components had an RLE of 13.2 years, compared to 8.9 years for those with ≥ 3 MS. Women had an RLE of 15.9 years with 0 MS components versus 11.4 years with ≥ 3 MS. RLE varied by cancer type, with the highest RLE in men seen in prostate cancer and in women in non-Hodgkin lymphoma. The lowest RLE for both sexes was in pancreatic cancer. The largest differences between 0 and ≥ 3 MS components were observed in non-Hodgkin lymphoma and the smallest in pancreatic cancer. Increased MS components were associated with reduced RLE in at least 8 cancer types for men and 9 for women. Prevention strategies targeting MS components could increase RLE in cancer patients.

**Supplementary Information:**

The online version contains supplementary material available at 10.1186/s12889-025-21437-9.

## Introduction

Metabolic syndrome (MS) forms a cluster of metabolic dysregulations including insulin resistance, dyslipidemia, central obesity, and hypertension. MS is significantly associated with an increased risk of developing diabetes and cardiovascular diseases [1]. Moreover, some studies [2–4] found an association between MS and a higher risk of liver, colorectal and bladder cancer in men; and endometrial, pancreatic, colorectal, ovarian and post-menopausal breast cancer in women.

MS is associated with a 1.5-fold increase in the risk of all-cause mortality [5]. Recent studies have examined MS and its components with cancer recurrence and overall mortality [6, 7]. The relationship between MS and cancer is complex; individual components of the MS are known risk factors for incident cancer disease, but it is not clear how the clustering of these components is linked to the development and progression of tumors, including cancer mortality. It seems self-evident that a condition characterized by multiple risk factors, like MS, will carry a greater risk for adverse clinical outcomes than will a single risk factor [6] The more components of MS present, the higher the mortality in cancer patients [8]. Contrarily, a study from Li W et al [9]. did not find an association between MS and cancer mortality. Thus, the association between MS and cancer mortality remains unclear [10].

However, from a clinical point of view, it is preferable to convey to patients their life expectancy. In this sense, this study focuses on the concepts remaining life expectancy (RLE) and life years lost (LYL). RLE after a cancer diagnosis provides a potentially more easily interpretable survival-based measure with real-world meaning, by quantifying a cancer’s impact over a lifetime horizon [11, 12].

Electronic health records (EHR) are a rich clinical data source that could be used to create individualized life expectancy predictions to identify cancer patients.

Studying the effect of MS as a risk factor on life expectancy is novel. The main aim of this study was to investigate the RLE, LYL and survival time of cancer cases according to the MS number of components. We investigated the effect of MS and the number of its components in a cohort of 13 cancer cases.

## Material and methods

### Data source and setting

Data were collected from the Information System for the Development of Research in Primary Care (SIDIAP) database, covering the period from January 1, 2008, to December 31, 2017. This database consists of the EHR of 286 primary healthcare centers (approximately 5.8 million individuals, 75% of the residents of Catalonia, Spain) [13]. The SIDIAP contains information on sociodemographic characteristics and clinical diagnoses, coded using the International Classification of Diseases, 10th revision (ICD-10), clinical parameters, laboratory tests outcomes and prescriptions and dispensations of medicines (using the Anatomical Therapeutic Chemical *(ATC)* Classification System) [14].

The cause of death was provided by the National Institute of Statistics (INE) [15], a Spanish public body responsible for the general coordination of the statistical services of the General Administration of the State. This data source was used to collect information on the cause of death among individuals in this study. The data contains the cause of death for each study participant who died during the follow-up. The cause of death is coded according to the official medical death certificate in Spain. For this study, the cause of death has been categorized as Cancer/Other causes.

### Study population and design

We designed a population-based cohort including incident cases of 13 cancer types. We included all participants aged 40 years or older who were diagnosed with their first incident (primary) cancer after January 1, 2008. We followed participants starting from the date of cancer diagnosis and until their death, until they were no longer part of the SIDIAP database (due to reason such as emigration), until they reached the age of 100 years, or until the end of the study period (December 31, 2017).

Patients were excluded from participation if two different cancer types were registered on the same date or they presented secondary cancers and metastases (6826 cases), if men were diagnosed with breast cancer (334 cases), or if there was a possible error in diagnosis (such as men with endometrial cancer or women with prostate cancer, 7 cases). The attrition rate was 3.9%.

Information regarding the study’s structure and the accuracy of the initial measurements has been documented in other publications [13, 16].

### Cancer definition

Cases of cancer were identified as individuals diagnosed with cancer for the first time between January 1, 2008, and December 31, 2017. We selected the most prevalent types of cancer with published evidence of association with MS such as colorectal ICD-10 codes C18 + C20), prostate (C61), liver (C22), bladder (C67), endometrial (C54), pancreas (C25) and breast (C50). Although few published data are available, we also included lung (C34) and kidney cancer (C64) because of their high prevalence. Finally, we included types of cancer for which little evidence is available but that which might also be associated with MS such as thyroid cancer (C73), Hodgkin lymphoma (C81), non-Hodgkin lymphoma (C82-85) and leukemia (C91-95).The analyses were done for any of these cancers combined or stratified by cancer type. Analyses for breast and endometrial cancers were stratified according to menopausal status (pre- and post-), guided by evidence suggesting different effects of obesity and estrogens during these two life stages [17].

### MS definition

As per criteria set by the American Heart Association/National Heart, Lung, and Blood Institute (AHA/NHLBI), a diagnosis of MS is established if an individual exhibits three or more of the following components: obesity, high blood pressure (HBP), reduced HDL cholesterol, elevated triglycerides (TG) and high glycemia [18]. We defined obesity as a body mass index (BMI) > 30 kg/m^2, serving as a measure of overall adiposity. Details of the definition of MS have been published elsewhere [19]. In cases where multiple measurements for a component were documented on the same day, we calculated the mean of these values.

For each individual, we assessed MS on the date of cancer diagnosis (based on prior history data) and classified individuals as having 0 MS components, 1 MS components, 2 MS components and ≥ 3 MS components.

### Covariables

We also extracted information on age (in years), sex, menopausal status, nationality (Spanish, non-Spanish); the MEDEA deprivation index (census tract-based deprivation index to identify socioeconomic status in urban areas) categorized in quintiles, with the first quintile representing the least deprived areas and the fifth quintile representing the most deprived areas, along with rural areas; smoking status (non-smokers, ex-smokers and current smokers); alcohol intake calculated in standard units (no alcohol, low and high consumption); dispensation of drugs such as hormonal replacement therapy among menopausal women, paracetamol, aspirin and ibuprofen (classified as yes/ no); presence of hepatitis (classified as yes/ no) and menopause status (classified as pre/post, ICD-10 code N95). For women lacking data regarding menopausal status, we considered being ≥ 50 years of age as indicative of post-menopausal status.

### Statistical analysis

We conducted a descriptive analysis of population, utilizing the mean (standard deviation) and median (interqualtile rage (IQR)) for numerical variables and percentages for categorical variables, stratified by the number of MS components.

With the Life Years Lost method [20] we estimated the LYL and RLE among those individuals with 0, 1, 2 and ≥ 3 MS components. This approach utilizes a Markov illness-death model combined with population life tables to estimate LYL and RLE at specific ages and calculate differences between groups. These calculations were based on age-specific mortality rates derived from population life tables and adjusted for competing risks using a multistate model. The method also allows for the decomposition of life expectancy into components attributable to different causes of death, providing a comprehensive view of the impact of metabolic syndrome on survival outcomes in cancer patients. We calculated these metrics at three different ages, using the median and the percentiles 25 and 75 at time of cancer diagnosis of the population. We calculated the total LYL of the individuals of 1, 2, ≥ 3 MS components (and the 95% confidence interval (CI)) compared with the individuals with 0 MS components. The LYL has been divided into two categories based on cause of death: due to cancer and due to other causes. We stratified the analyses by sex and by each cancer type.

Kaplan–Meier curves and log-rank tests were performed according to MS for total cancer and for each cancer type, and stratified by sex (gynecological cancers were stratified by menopausal status at the time of cancer diagnosis).

All statistical analyses were conducted using R version 4.4.0 and the R package lillies [21].

## Results

This study included 183,364 individuals with a cancer diagnosis, of whom 55,603 died during the follow-up. The distribution of individuals by cancer type was as follows: 37,926 breast (20.7%); 36,155 colorectal (19.7%); 30,879 prostate (16.8%); 20,792 bladder (11.3%); 20,337 lung (11.1%); 6,939 leukemia (3.8%); 6,826 kidney (3.7%); 5,735 liver (3.1%); 5,391 pancreatic (2.9%); 5,382 endometrial (2.9%); 3,616 non-Hodgkin lymphoma (2.0%); 2,705 thyroid (1.5%) and 681 Hodgkin lymphoma (0.4%).

Baseline characteristics of all individuals categorized by the numbers of MS components are summarized in Table 1. During the study period, 19.5% of individuals with 0 MS components at baseline died. Mortality rates increased with the number of MS components: 29.9% for those with 1 component, 33.3% for those with 2 components, and 32.1% for those with 3 or more components. Cancers with the highest mortality during the follow up were pancreas (72.6%), lung (67.5%), and liver (66.8%), while the cancers with the lowest mortality were thyroid (7.8%), breast (12.6%) and prostate (18.8%). The median follow-up time was 3.15 years (IQR: 1.18 years to 5.89 years). The median follow-up time was longer for patients with 0 MS components (4.62 years, IQR 1.99 to 7.29 years) and shorter for those with ≥ 3 MS components (2.50 years, IQR 0.92 to 4.95 years). The study included 79,937 women and 103,427 men. During the follow-up period, 23.9% of the women and 35.3% of the men died. Most participants were never smokers (31.9%), %), and 16.3% were in the least deprived category of the MEDEA deprivation index. Additionally, 73.6% of the women were post-menopausal at the time of diagnosis.

**Table 1:** Characteristics of the study population at baseline

	All Cases n(%)	Cases who survived during the follow-up	Cases who died during the follow-up
N total	183,364	127,761	55,603
**Age** mean (SD)	67.9 (12.4)	65.6 (11.9)	73.3 (12.1)
Median (IQR)	68.2 (58.9–77.6)	65.8 (57.0–74.5)	75.2 (64.9–82.5)
**Sex**			
Men	103,427 (56.4)	66,943 (52.4)	36,484 (65.6)
Women	79,937 (43.6)	60,818 (47.6)	19,119 (34.4)
**Metabolic Syndrome**			
No component	27,761 (15.1)	22,352 (17.5)	5409 (9.7)
1 component	42,822 (23.4)	30,038 (23.5)	12,784 (23.0)
2 components	40,561 (22.1)	27,059 (21.2)	13,502 (24.3)
≥ 3 components	72,220 (39.4)	48,312 (37.8)	23,908 (43.0)
**Cancer Type**			
Colon_Rectal	36,155 (19.7)	25,140 (19.7)	11,015 (19.8)
Liver	5735 (3.1)	1905 (1.5)	3830 (6.9)
Pancreas	5391 (2.9)	1478 (1.2)	3913 (7.0)
Breast	37,926 (20.7)	33,154 (26.0)	4772 (8.6)
Endometrial	5382 (2.9)	4330 (3.4)	1052 (1.9)
Bladder	20,792 (11.3)	14,802 (11.6)	5990 (10.8)
Kidney	6826 (3.7)	5169 (4.0)	1657 (3.0)
Prostate	30,879 (16.8)	25,060 (19.6)	5819 (10.5)
Hodgkin	681 (0.4)	536 (0.4)	145 (0.3)
Non_Hodgkin	3616 (2.0)	2645 (2.1)	971 (1.7)
Leukemia	6939 (3.8)	4433 (3.5)	2506 (4.5)
Lung	20,337 (11.1)	6614 (5.2)	13,723 (24.7)
Thyroid	2705 (1.5)	2495 (2)	210 (0.4)
**Follow-up time** Median (IQR)	3.15 (1.18–5.89)	4.17 (1.89–6.75)	1.36 (0.52–3.12)
Follow-up time (No MS component) Median (IQR)	4.62 (1.99–7.29)	5.34 (2.78–7.76)	1.79 (0.74–3.82)
Follow-up time (1 MS component) Median (IQR)	3.62 (1.40–6.43)	4.69 (2.26–7.18)	1.56 (0.61–3.52)
Follow-up time (2 MS components) Median (IQR)	3.10 (1.14–5.82)	4.20 (1.93–6.74)	1.38 (0.51–3.23)
Follow-up time (≥ 3 MS components) Median (IQR)	2.50 (0.92–4.95)	3.42 (1.51–5.78)	1.18 (0.44–2.74)
**Nationality**			
Spanish	178,320 (97.2)	123,718 (96.8)	54,602 (98.2)
Non-Spanish	5044 (2.8)	4043 (3.2)	1001 (1.8)
**MEDEA index**			
Quintile 1	29,808 (16.3)	23,708 (18.6)	6100 (11.0)
Quintile 2	26,416 (14.4)	20,363 (15.9)	6053 (10.9)
Quintile 3	25,152 (13.7)	19,107 (15.0)	6045 (10.9)
Quintile 4	23,684 (12.9)	17,935 (14)	5749 (10.3)
Quintile 5	20,381 (11.1)	14,877 (11.6)	5504 (9.9)
Rural	33,972 (18.5)	22,887 (17.9)	11,085 (19.9)
Missings	23,951 (13.1)	8884 (7)	15,067 (27.1)
**Smoking status**			
Never smoker	58,553 (31.9)	43,158 (33.8)	15,395 (27.7)
Ex-smoker	20,909 (11.4)	14,649 (11.5)	6260 (11.3)
Smoker	19,789 (10.8)	13,513 (10.6)	6276 (11.3)
Missings	84,113 (45.9)	56,441 (44.2)	27,672 (49.8)
**Alcohol intake**			
No consumption	58,951 (32.1)	42,253 (33.1)	16,698 (30.0)
Low consumption	34,401 (18.8)	25,350 (19.8)	9051 (16.3)
High consumption	3569 (1.9)	2355 (1.8)	1214 (2.2)
Missings	86,443 (47.1)	57,803 (45.2)	28,640 (51.5)
**Hormonal therapy (women postmenopausia)**			
No consumption	54,913 (93.3)	38,873 (92.9)	16,040 (94.4)
Consumption	3916 (6.7)	2962 (7.1)	954 (5.6)
**Paracetamol**			
No consumption	129,248 (70.5)	94,252 (73.8)	34,996 (62.9)
Consumption	54,116 (29.5)	33,509 (26.2)	20,607 (37.1)
**Acetylsalicylic acid (ASA)**			
No consumption	151,386 (82.6)	109,249 (85.5)	42,137 (75.8)
Consumption	31,978 (17.4)	18,512 (14.5)	13,466 (24.2)
**Ibuprofen**			
no consumption	156,781 (85.5)	109,151 (85.4)	47,630 (85.7)
consumption	26,583 (14.5)	18,610 (14.6)	7973 (14.3)
**Chronic Hepatitis**			
No hepatitis	182,007 (99.3)	127,170 (99.5)	54,837 (98.6)
Hepatitis B	258 (0.1)	143 (0.1)	115 (0.2)
Hepatitis C	1078 (0.6)	440 (0.3)	638 (1.1)
Other/unspecified hepatitis	21 (0.0)	8 (0)	13 (0.0)
**Menarche age** mean (SD)	12.6 (1.6)	12.6 (1.6)	12.7 (1.6)
Median (IQR)	13 (12–14)	13 (12–14)	13 (12–14)
Missings n(%)	63,642 (79.6)	46,123 (75.8)	17,519 (91.6)
**Menopause**			
No	21,108 (26.4)	18,983 (31.2)	2125 (11.1)
Yes	58,829 (73.6)	41,835 (68.8)	16,994 (88.9)
**Primary care visits between 2 and 4 years before data index** mean (SD)	14.2 (17.4)	13.4 (15.8)	16.1 (20.4)
Median (IQR)	9 (1–21)	9 (2–19)	10 (1–24)

The median age at cancer diagnosis among all the individuals in the study was 68 years. In women with a cancer diagnosis at 68 years and 0 MS components, the RLE was 15.95 years (95% CI: 15.37–16.53), 13.79 years (95% CI: 13.48–14.21) for 1 MS component, 12.36 years (95% CI: 12.02–12.64) for 2 MS components and 11.38 years (95% CI: 11.16–11.62) for ≥ 3 MS components. The RLE decreased as the number MS components increased. The LYL for women with ≥ 3 MS components, compared with women with 0 MS components was 4.57 years (95% CI 3.92–5.12). The LYL increased as the number of MS components increased (Table 2).

**Table 2:** Remaining life expectancy when the cancer diagnosis was at 68 years

	**0 MS**	**1 MS**	**2 MS**	**≥ 3 MS**
**Women**				
Remaining life expectancy	15.95 (15.37–16.53)	13.79 (13.48- 14.21)	12.36 (12.02–12.64)	11.38 (11.16–11.62)
Life Years Lost	Ref	2.17 (1.38–2.75)	3.59 (2.85–4.24)	4.57 (3.92–5.12)
- Due to Cancer	Ref	0.93 (-0.01–1.72)	3.30 (2.38–4.05)	3.68 (3.01–4.56)
- Due to Other Causes	Ref	1.24 (0.43–1.91)	0.29 (-0.43–0.82)	0.89 (0.24–1.36)
**Men**				
Remaining life expectancy	13.23 (12.78–13.68)	10.89 (10.67–11.15)	10.13 (9.89–10.38)	8.92 (8.71–9.08)
Life Years Lost	Ref	2.34 (1.88–2.81)	3.10 (2.62–3.57)	4.31 (3.88–4.74)
- Due to Cancer	Ref	1.37 (0.71–2.09)	2.21 (1.60–2.98)	3.65 (3.05–4.33)
- Due to Other Causes	Ref	0.97 (0.40–1.32)	0.88 (0.35–1.28)	0.66 (0.17–1.06)

^*^95% confidence intervals based on 100 bootstrap iterations

In men with cancer diagnosis at 68 years and 0 MS components, the RLE was 13.23 years (95% CI: 12.78–13.68), 10.89 years (95% CI: 10.67–11.15) for 1 MS component, 10.13 years (95% CI: 9.89–10.38) for 2 MS components and 8.92 years (95% CI: 8.71–9.08) for ≥ 3 MS components. The RLE decreased as the number of MS increased. The LYL of men with 1 MS component, compared to those with 0 MS components was 2.34 years (95% CI 1.88–2.81), 1.37 years (95% CI: 0.71–2.09). The LYL increased as the number of MS components increased (Table 2).

**Fig. 1:**
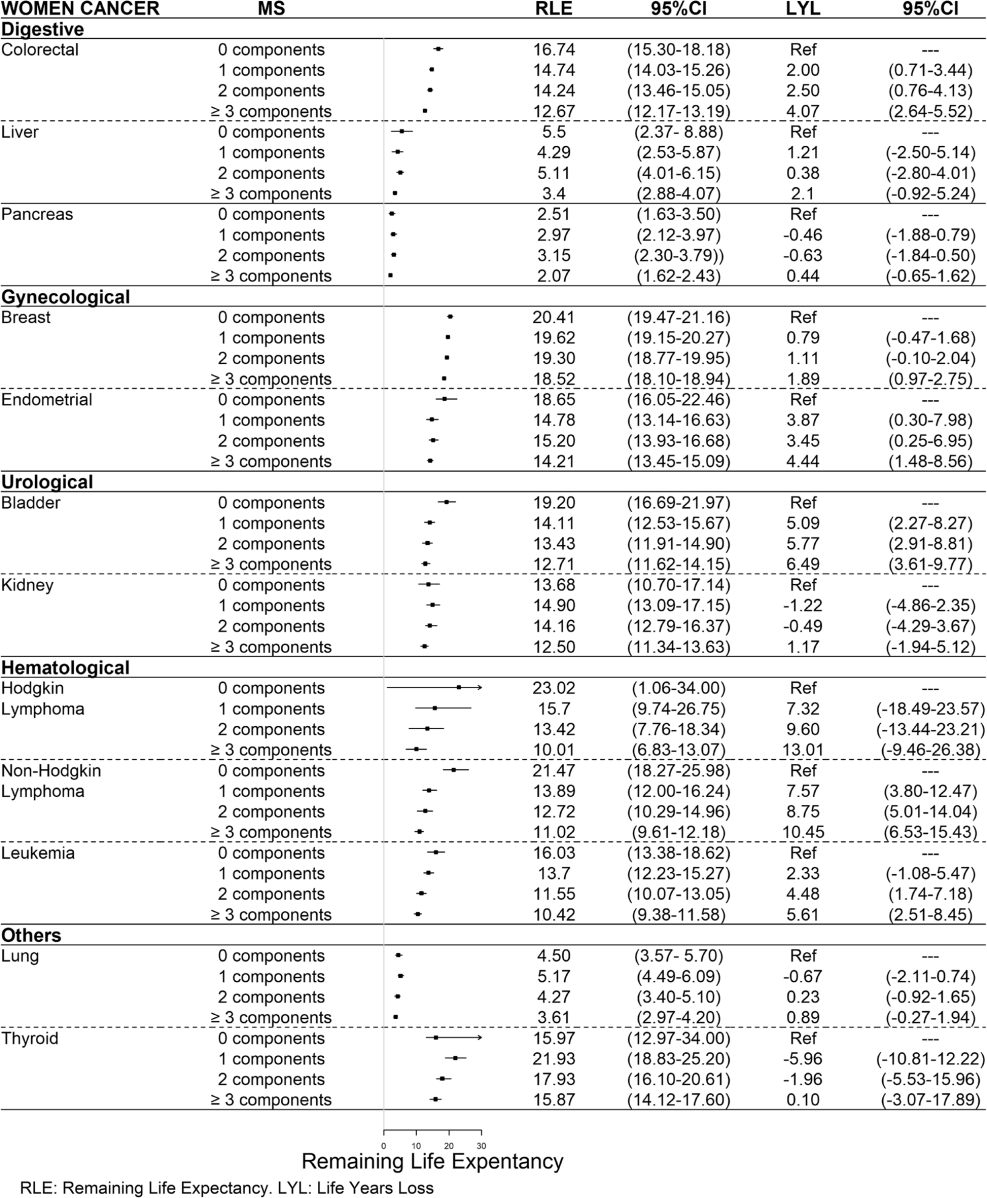
Remaning life expectancy (and Life of Years Lost) by cancer type in women

**Fig. 2:**
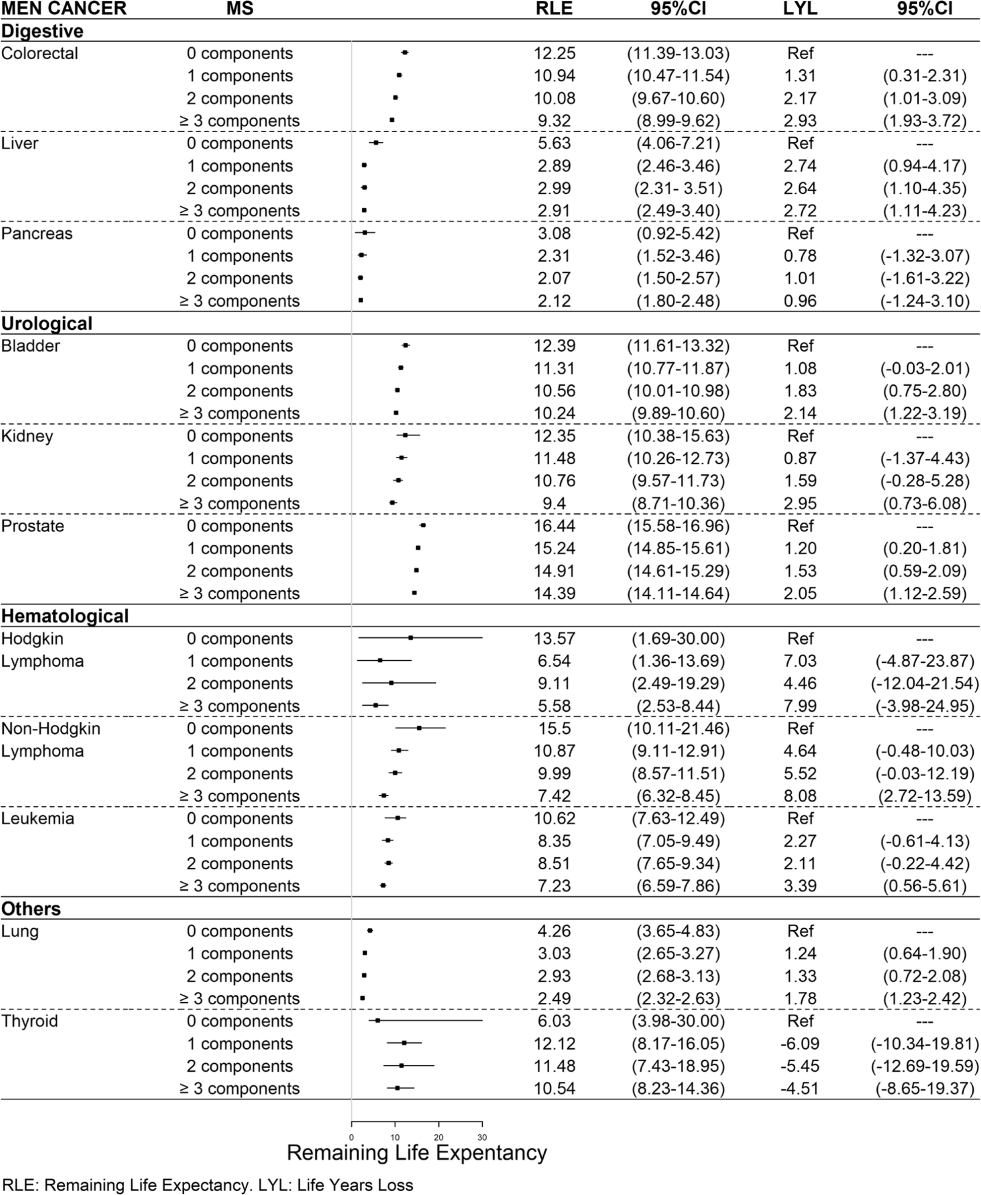
Remaning life expectancy (and Life of Years Lost) by cancer type in men

Figures 1 and 2 show the RLE in women and men diagnosed with cancer at 68 years of age, depending on the type of cancer and the number of MS components. In general, from 1 MS component onwards, there was a decrease in RLE as the number of MS components increased. Exceptions for this trend were observed in pancreas, kidney and thyroid cancer in women and Hodgkin lymphoma and thyroid cancer in men. In pancreatic cancer in women, the RLE was similar regardless of the number of MS components. In kidney cancer in women, the RLE for those with 0 MS components was lower compared to those with 1 MS component. In thyroid cancer in women, the RLE of individuals with 0 MS components was lower compared to those with ≥ 1 MS components. In Hodgkin lymphoma in men, the RLE was lower in the participants with 2 MS components. For thyroid cancer in men, the RLE was lower in those with 0 MS components.

RLE was higher in both women and men when cancer was diagnosed at 59 years old (20.8 and 15.3 in women with 0 and ≥ 3 MS components respectively, and 15.3 and 10.4 in men with 0 and ≥ 3 MS components respectively) and lower when cancer was diagnosed at 78 years old (10.2 and 7.0 in women with 0 and ≥ 3 MS components respectively, and 8.8 and 5.9 in men with 0 and ≥ 3 MS components respectively) (Supplementary Table 1–2).

**Fig. 3:**
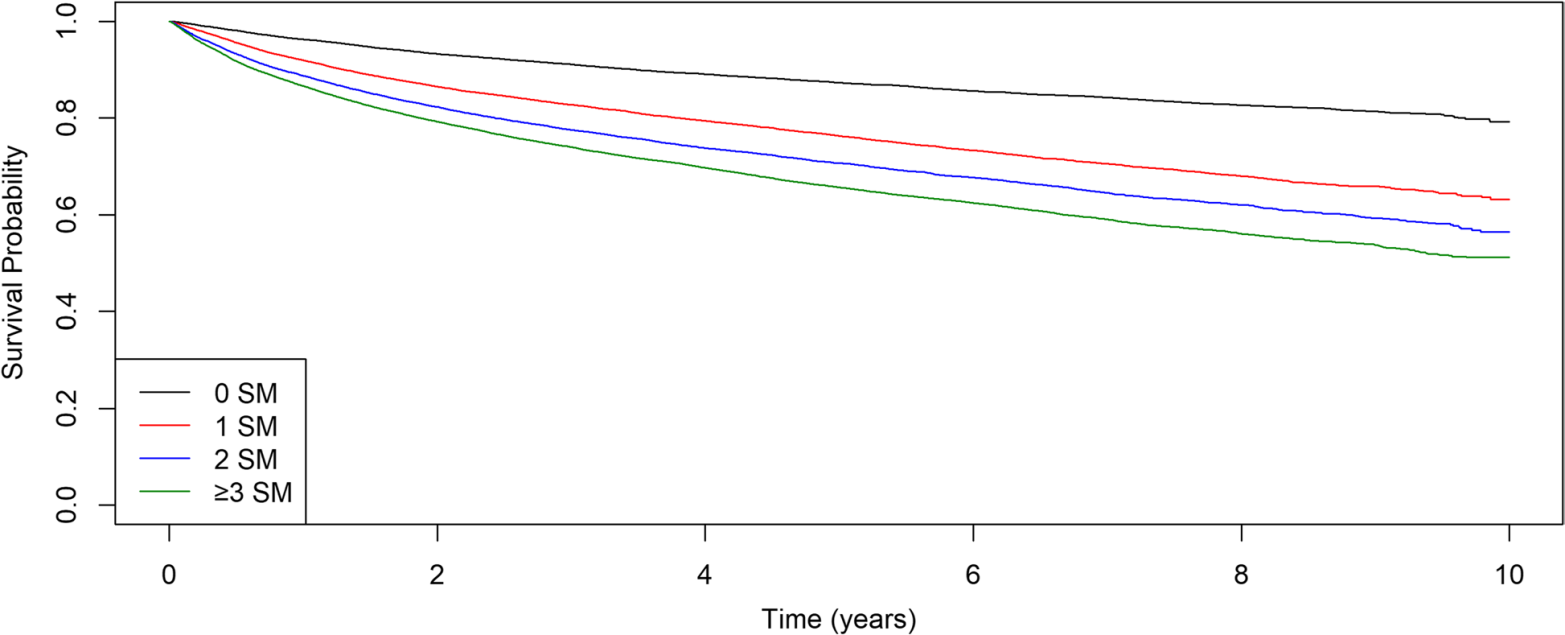
Women with Cancer. Kaplan-Meier Curve by MS

**Fig. 4:**
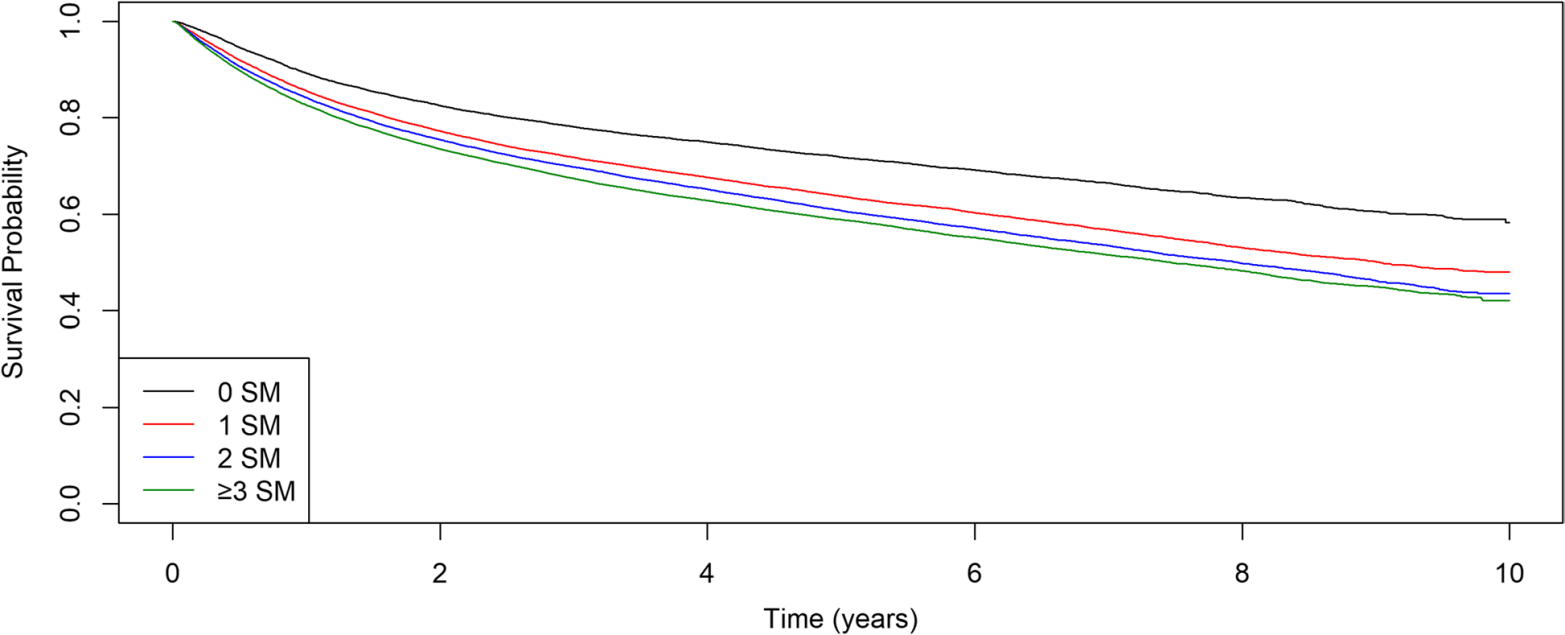
Men with Cancer. Kaplan-Meier Curve by MS

The 5-years survival rate was higher with fewer MS components. In men with cancer, the 5-year survival rate was 71.8% in the group with 0 MS components, compared to 58.9% in the group with ≥ 3 MS components. In women with cancer, the 5-year survival rate was 87.3% in the group with 0 MS components, compared to 65.7% in the group with ≥ 3 MS components (Figs. 3 and 4, Supplementary Tables 3–4, Supplementary Figs. 26–27).

Regarding cancer type, using Kaplan–Meier analysis, 5-year survival rates generally decreased as the number of associated components increases. However, no statistically significant relationship was observed between a higher number of MS and shorter survival in some specific cancers: i) in women with pre-menopausal endometrial cancer, individuals with 2 MS components had a higher survival at 5 years than individuals with 1 MS components; ii) in men with Hodgkin lymphoma, the survival at 5 years in individuals with 1 MS component was lower than the survival in the individuals with 2 MS components (Supplementary Figs. 28–54).

Supplementary Figs. 3–25 illustrate the RLE in men and women with cancer diagnosis between the ages 59 and 78, stratified by cancer type and the number of MS components. RLE decreased as the number of components of MS increased in the majority of cancer types. In general, the observed pattern aligns with global cancer trends.

## Discussion

In this population-based cohort, we found that MS, and a higher number of MS components were associated with a lower RLE for cancer, specifically in 8 of 11 cancer types in men and 9 of 12 in women. These effects were independent of the age at cancer diagnosis. We observed consistent results using the median age of cancer diagnosis (68 years) and the 25th and 75th percentiles (59 and 78 years) in the LYL and RLE analyses. Moreover, all cancer cases exhibited lower survival rates in patients with 1, 2, or ≥ 3 MS components compared to those with 0 MS components.

### Cancer mortality

Recent articles have reported a significant relationship between MS and mortality in cancer patients [10, 22, 23]. Similar to our findings, the Watanabe study [22], as well as other studies such as J-MICC [23] and NHANES III [10], reported that the risk of cancer mortality increases with the number of MS components. Consequently, the addition of MS components appears to increase cancer mortality risk, in contrast to the presence of individual components, which is associated with a lower mortality risk [24, 25].

When examining associations by specific cancer types, despite methodological differences, our results align with those several studies. In other studies MS showed worse survival in non-Hodgkin lymphoma, and for men, in kidney, prostate, and lung [26] cancer, which is consistent with our study. We found no studies examining MS and mortality in Hodgkin lymphoma or leukemia. Nevertheless, individual components of MS, such as obesity and low LDL, have been linked to higher mortality in Hodgkin lymphoma [27] and HBP have been associated with an increased of leukemia mortality. Similarly, studies lack sufficient follow-up to confirm the impact of MS on thyroid cancer survival, as noted by Li et al. [28].

We evaluated LYL differences among cancer patients based on MS presence, finding shorter LYL for patients with 1, 2, or ≥ 3 MS components compared to those with 0 MS components. To the best of our knowledge, no studies have specifically analyze LYL and MS in cancer patients, although LYL has been used in other conditions. LYL provides conservative estimates by accounting for age at onset [29]. Additionally, LYL is a valuable tool for assessing the impact of diseases on life expectancy in clinical and epidemiological research [10, 11].

### Sex-specific differences

In the study of Watanabe et al. [22] MS was positively associated with cancer mortality in women but not in men Moreover, in our study differences in RLE and Kaplain-Meier curves based on sex were observed, with better survival outcomes in women. Our findings indicate that MS is associated with a decrease in RLE across various cancer types in men including colorectal, liver, pancreatic, bladder, kidney, and prostate cancers, as weel as leukemia, and Hodgkin and non-Hodgkin lymphomas. For women, the affected cancers included colorectal, liver, breast, endometrial, and bladder cancers, along with leukemia, and Hodgkin and non-Hodgkin lymphomas according to other published works [27, 30–40

RLE is generally greater in women than in men, reflecting findings from other studies [41, 42] that suggest this disparity can not be fully explained by differences in cancer stage, grade, or treatment. Lifestyle factors and immunological differences contribute to these sex-based survival disparities.

In gynecological cancers (breast and endometrial), we observed a clear trend of decreased survival with an increased number of MS components in post-menopausal women. For pre-menopausal breast cancer, a similar but less pronounced trend was noted. However, in pre-menopausal endometrial cancer, survival increased with 2 MS components but decreased with 1 MS component according to the Kokts-Porietis et al. study [38].

### Comparative survival analysis: LYL, RLE, and Kaplan–Meier methods

Compared with Cox and Kaplan–Meier methods, RLE provides a more straightforward interpretation by directly quantifying survival time between different groups, aiding clear communication with clinicians and patients, and offering a clearer understanding of the impact of various factors on overall survival. RLE focuses on patient-centered outcomes by emphasizing the total lifespan, which is often more relevant to patients than relative measures of risk (like HRs). Additionally, RLE provides absolute measures of survival time, which can be more informative in clinical decision-making, while Kaplan–Meier plots provide valuable visual information and are widely used.

The Kaplan–Meier plot results are very similar to those from the RLE analysis. Several studies using Kaplan–Meier methods have found that patients with ≥ 3 MS components have lower survival rates than those with 0 MS components, particularly in cases of colorectal [43], breast [37], bladder [39], prostate [31], kidney [30] cancer, and non-Hodgkin lymphoma [26]. Although some studies show slightly different survival rates, the overall pattern of reduced survival in patients with ≥ 3 MS components compared to those with 0 MS components is consistent with our findings (Supp. Figures 26–54). Kaplan–Meier analyses illustrate survival trajectories over the 10-year follow-up, showing how survival probabilities evolve. In contrast, the main analyses summarize the impact of RLE and LYL across the lifespan. Together, they provide complementary perspectives: Kaplan–Meier analyses focus on short- to medium-term survival, while RLE and LYL offer a long-term overview [44].

### Strengths and limitations

A key strength of this study is its large, population-based sample from the SIDIAP and INE databases. SIDIAP includes approximately 75% of the Catalonian population, ensuring high representativeness [13], and INE accurately provides the causes of death of the deceased participants [15].

This study also contributes novel insights by examining MS and survival in underexplored cancer types, including Hodgkin lymphoma, leukemia, and thyroid cancer, expanding the current literature on this topic. Our study uniquely applies RLE analyses to MS, a method not used in previous research, making our findings novel and impactful.

Our study has several limitations. One is the assumption of a linear relationship between the cumulative number of MS components and cancer mortality risk, while the AHA/NHLBI criteria emphasize the binary categorization of MS. At the beginning of the follow-up, some individuals lacked data on specific MS components, we assumed that missing data indicated normal levels, potentially introducing misclassification bias. Additionally, the RLE analysis only adjusts for age and excludes confounders like tobacco use, alcohol consumption, socioeconomic status, nationality, physical activity, parity, and treatment. Notably, our data on tobacco and alcohol usage exhibit a considerable proportion of missing entries, especially at the beginning of the follow-up. However, this does not alter or introduce bias into the analyses, as the LYL and RLE analyses do not adjust for covariables. Obesity was classified using BMI rather than abdominal fat, aligning with the World Health Organization (WHO) [45]. We also lacked data on cancer treatment, which may influence survival outcomes. Finally, the RLE estimates should be interpreted cautiously and not extrapolated as the number of years left to live for cancer patients in general. The cohort primarily includes survivors diagnosed between 2008 and 2017, a period of high early mortality post-diagnosis, and predictions may be less accurate for rare cancers.

## Conclusion

The prevalence of MS and cancer is increasing worldwide [46, 47] while recent reductions in cancer mortality reflect progress in prevention, though mortality rates remain high. Recognizing the significance of MS in cancer mortality is crucial for understanding its impact on increasing RLE.

MS is becoming increasingly prevalent among young adults due to changes in lifestyle, diet, and socioeconomic environment. While healthy lifestyles may reduce MS incidence, further research is needed to determine if interventions targeting MS will improve life expectancy. The role of MS in cancer survivors is an emerging research topic [48].

In conclusion, MS is significantly linked to reduced RLE, with more MS components statistically associated with decreased RLE in 8 cancer types in men and 9 in women. Women generally show higher RLE and better survival than men. This study provides compelling evidence that informs cancer prevention and management strategies for individuals with MS. Our findings suggest that implementing preventive measures targeting specific MS components could potentially increase RLE in cancer patients.

## Supplementary Information

Supplementary Material 1.

## Data Availability

The data utilized for analysis in this study were sourced from EHR within the primary healthcare clinical history and are only available for the participating researchers, in accordance with current European and national laws. Thus, the distribution of the data is not allowed. However, researchers from public institutions can request data from SIDIAP. Further information is available online (https://www.sidiap.org/index.php/menu-solicitudes-en/application-proccedure).
