# Supplementary material for: The effect between metabolic syndrome and life expectancy after cancer diagnosis: Catalan cohort study
Source: BMC Public Health. 2025 Jan 16;25:178. doi: 10.1186/s12889-025-21437-9 (PMC11740694; doi:10.1186/s12889-025-21437-9)
Supplement: Supplementary file 1 — Supplementary Material 1. [file 12889_2025_21437_MOESM1_ESM.docx]

**Supp. Table 1.**

**Women with Cancer.** Remaining life expectancy with cancer diagnosed at ages 59 and 78

| **Diagnosis at 59 years** | **0 MS** | **1 MS** | **2 MS** | **≥3 MS** |
| --- | --- | --- | --- | --- |
| Remaining life expectancy | 20.81  (20.25-21.39) | 18.26  (17.87-18.83) | 17.03  (16.56-17.39) | 15.28  (14.88-15.57) |
| Life Years Lost | Ref | 2.55  (1.85-3.23) | 3.79  (3.09-4.68) | 5.53  (4.91-6.24) |
| - Due to Cancer | Ref | 1.52  (0.65-2.36) | 3.42  (2.47-4.36) | 4.50  (3.70-5.42) |
| - Due to Other Causes | Ref | 1.03  (0.30-1.57) | 0.37  (-0.30-0.94) | 1.04  (0.47-1.59) |
|  |  |  |  |  |
| **Diagnosis at 78 years** | **0 MS** | **1 MS** | **2 MS** | **≥3 MS** |
| Remaining life expectancy | 10.23  (9.74-10.68) | 8.65  (8.37-8.86) | 7.73  (7.51-7.94) | 6.95  (6.81-7.10) |
| Life Years Lost | Ref | 1.57  (1.04-2.18) | 2.49  (1.94-3.15) | 3.27  (2.77-3.84) |
| - Due to Cancer | Ref | 0.60  (-0.10-1.29) | 2.23  (1.38-2.92) | 2.68  (1.96-3.37) |
| - Due to Other Causes | Ref | 0.97  (0.39-1.56) | 0.26  (-0.29-0.77) | 0.59  (0.05-1.16) |

*95% confidence intervals based on 100 bootstrap iterations

**Supp Table 2.**

**Men with Cancer.** Remaining life expectancy with cancer diagnosed at ages 59 and 78

| **Diagnosis at 59 years** | **0 MS** | **1 MS** | **2 MS** | **≥3 MS** |
| --- | --- | --- | --- | --- |
| Remaining life expectancy | 15.30  (14.85-15.74) | 13.07  (12.76-13.37) | 11.99  (11.69-12.33) | 10.42  (10.19-10.65) |
| Life Years Lost | Ref | 2.23  (1.78-2.82) | 3.31  (2.66-3.81) | 4.88  (4.35-5.43) |
| - Due to Cancer | Ref | 1.24  (0.44-2.03) | 2.15  (1.39-2.80) | 3.79  (3.00-4.51) |
| - Due to Other Causes | Ref | 0.99  (0.43-1.50) | 1.15  (0.67-1.69) | 1.09  (0.58-1.59) |
|  |  |  |  |  |
| **Diagnosis at 78 years** | **0 MS** | **1 MS** | **2 MS** | **≥3 MS** |
| Remaining life expectancy | 8.76  (8.36-9.06) | 7.06  (6.84-7.20) | 6.58  (6.42-6.75) | 5.87  (5.76-6.01) |
| Life Years Lost | Ref | 1.70  (1.34-2.13) | 2.18  (1.76-2.48) | 2.88  (2.53-3.22) |
| - Due to Cancer | Ref | 1.03  (0.52-1.60) | 1.47  (0.95-2.01) | 2.35  (1.82-2.92) |
| - Due to Other Causes | Ref | 0.67  (0.15-1.12) | 0.71  (0.14-1.24) | 0.53  (0.02-1.03) |

*95% confidence intervals based on 100 bootstrap iterations

**Supp Table 3.** 5 years survival rate by cancer type in women

| Cancer | 0 MS | 95% CI | 1 MS | 95% CI | 2 MS | 95% CI | ≥3 MS | 95% CI |
| --- | --- | --- | --- | --- | --- | --- | --- | --- |
| Global | 87.3 | 86.7-87.9 | 76.3 | 75.6-77.0 | 70.7 | 69.8-71.5 | 65.7 | 65.0-66.3 |
| Colorectal | 79.2 | 77.3-81.2 | 69.7 | 67.9-71.4 | 67.5 | 65.6-69.4 | 62.5 | 61.1-64.0 |
| Liver | 45.1 | 36.8-55.3 | 31.2 | 26.6-36.7 | 25.4 | 21.0-30.7 | 18.6 | 15.5-22.2 |
| Pancreas | 23.4 | 17.0-32.2 | 23.3 | 19.1-28.4 | 16.4 | 13.3-20.4 | 13.9 | 11.7-16.4 |
| Breast Pre | 95.3 | 94.7-95.9 | 94.7 | 93.8-95.6 | 93.3 | 91.8-94.9 | 92.5 | 90.7-94.3 |
| Breast Post | 90.5 | 89.4-91.6 | 83.2 | 82.1-84.3 | 81.8 | 80.5-83.1 | 78.8 | 77.8-79.9 |
| Endo Pre | 90.6 | 87.3-94.0 | 88.2 | 84.0-92.5 | 94.1 | 90.4-98.1 | 90.3 | 85.9-94.9 |
| Endo Post | 83.8 | 79.6-88.3 | 75.9 | 72.6-79.5 | 74.0 | 70.6-77.5 | 73.2 | 71.1-75.4 |
| Bladder | 86.2 | 82.6-89.9 | 67.1 | 63.5-70.9 | 62.3 | 58.3-66.5 | 62.4 | 59.4-65.5 |
| Kidney | 80.6 | 75.7-85.8 | 74.9 | 70.9-79.2 | 72.5 | 68.0-77.4 | 68.3 | 65.0-71.8 |
| Hodgkin | 91.7 | 84.7-99.2 | 85.7 | 75.6-97.2 | 67.8 | 55.4-83.1 | 61.1 | 50.5-73.9 |
| non-Hodgkin | 91.8 | 88.8-95.0 | 74.6 | 70.1-79.4 | 74.4 | 69.4-79.8 | 62.7 | 58.5-67.3 |
| Leukemia | 79.7 | 75.4-84.2 | 69.5 | 65.7-73.5 | 61.9 | 57.9-66.1 | 56.0 | 53.1-59.2 |
| Lung | 40.3 | 36.3-44.8 | 34.8 | 31.6-38.4 | 29.0 | 25.5-32.9 | 26.0 | 23.2-29.1 |
| Thyroids | 98.2 | 96.9-99.5 | 96.6 | 94.9-98.3 | 92.8 | 89.8-95.8 | 88.2 | 85.0-91.4 |

**Supp Table 4.** 5 years survival rate by cancer type in men

| Cancer | 0 MS | 95% CI | 1 MS | 95% CI | 2 MS | 95% CI | ≥3 MS | 95% CI |
| --- | --- | --- | --- | --- | --- | --- | --- | --- |
| Global | 71.8 | 70.9-72.7 | 63.7 | 63.0-64.4 | 60.7 | 60.0-61.4 | 58.9 | 58.3-59.4 |
| Colorectal | 72.6 | 70.6-74.6 | 66.2 | 64.6-67.8 | 62.7 | 61.1-64.3 | 62.3 | 61.1-63.6 |
| Liver | 34.9 | 29.4-41.5 | 23.6 | 20.4-27.2 | 26.2 | 23.1-29.7 | 22.3 | 20.0-25.0 |
| Pancreas | 27.3 | 21.7-34.4 | 20.4 | 16.5-25.2 | 16.4 | 13.3-20.2 | 14.4 | 12.2-17.0 |
| Bladder | 81.4 | 79.4-83.4 | 70.5 | 68.9-72.2 | 66.8 | 65.2-68.5 | 68.1 | 66.8-69.4 |
| Kidney | 82.0 | 78.4-85.7 | 75.9 | 72.9-79.1 | 70.0 | 66.6-73.6 | 69.4 | 66.8-72.0 |
| Prostate | 88.8 | 87.7-90.0 | 81.7 | 80.8-82.7 | 80.3 | 79.3-81.3 | 80.8 | 79.9-81.7 |
| Hodgkin | 90.4 | 84.5-96.7 | 77.8 | 68.8-87.9 | 78.1 | 67.6-90.2 | 71.1 | 62.7-80.5 |
| non-Hodgkin | 87.7 | 84.0-91.6 | 73.3 | 68.8-78.1 | 68.1 | 63.0-73.7 | 60.5 | 56.1-65.2 |
| Leukemia | 76.4 | 72.1-80.9 | 61.1 | 57.4-65.0 | 57.6 | 53.9-61.7 | 54.6 | 51.7-57.7 |
| Lung | 31.7 | 29.4-34.1 | 22.4 | 20.9-24.0 | 20.9 | 19.4-22.5 | 18.6 | 17.4-19.7 |
| Thyroids | 91.3 | 86.2-96.7 | 86.6 | 80.5-93.2 | 85.2 | 78.2-92.8 | 83.2 | 77.2-89.6 |

**Supp Figure 1.** Women. Cancer. Remaining Life Expentancy.

Cancer diagnosis between 59 and 78 years.
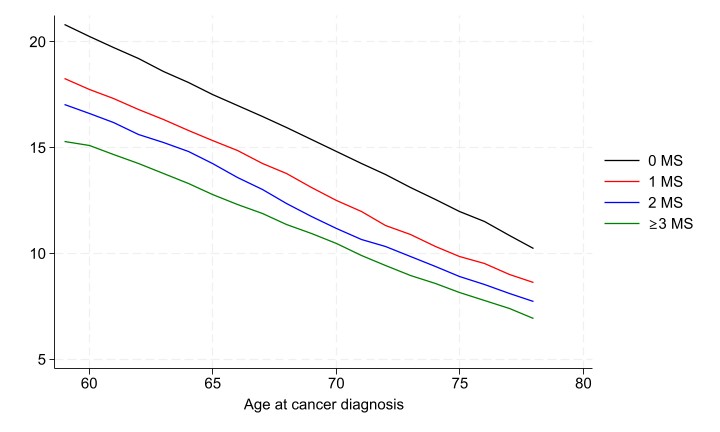


Remaing Life Expectancy

**Supp Figure 2.** Men. Cancer. Remaining Life Expentancy.

Cancer diagnosis between 59 and 78 years.
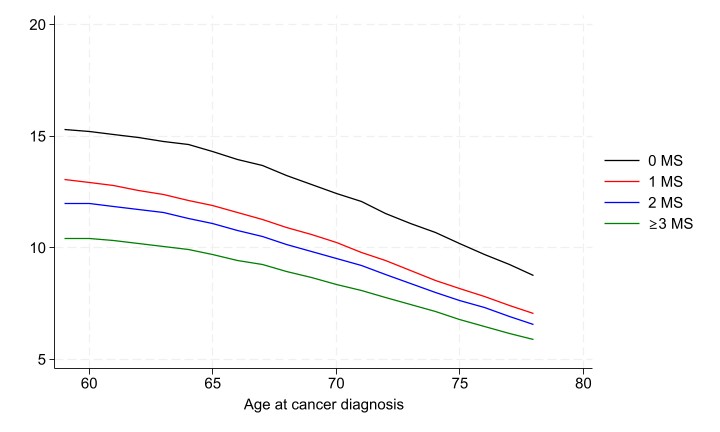


Remaing Life Expectancy

**Supp Figure 3.** Women. Colorectal cancer. Remaining Life Expentancy.

Cancer diagnosis between 59 and 78 years.


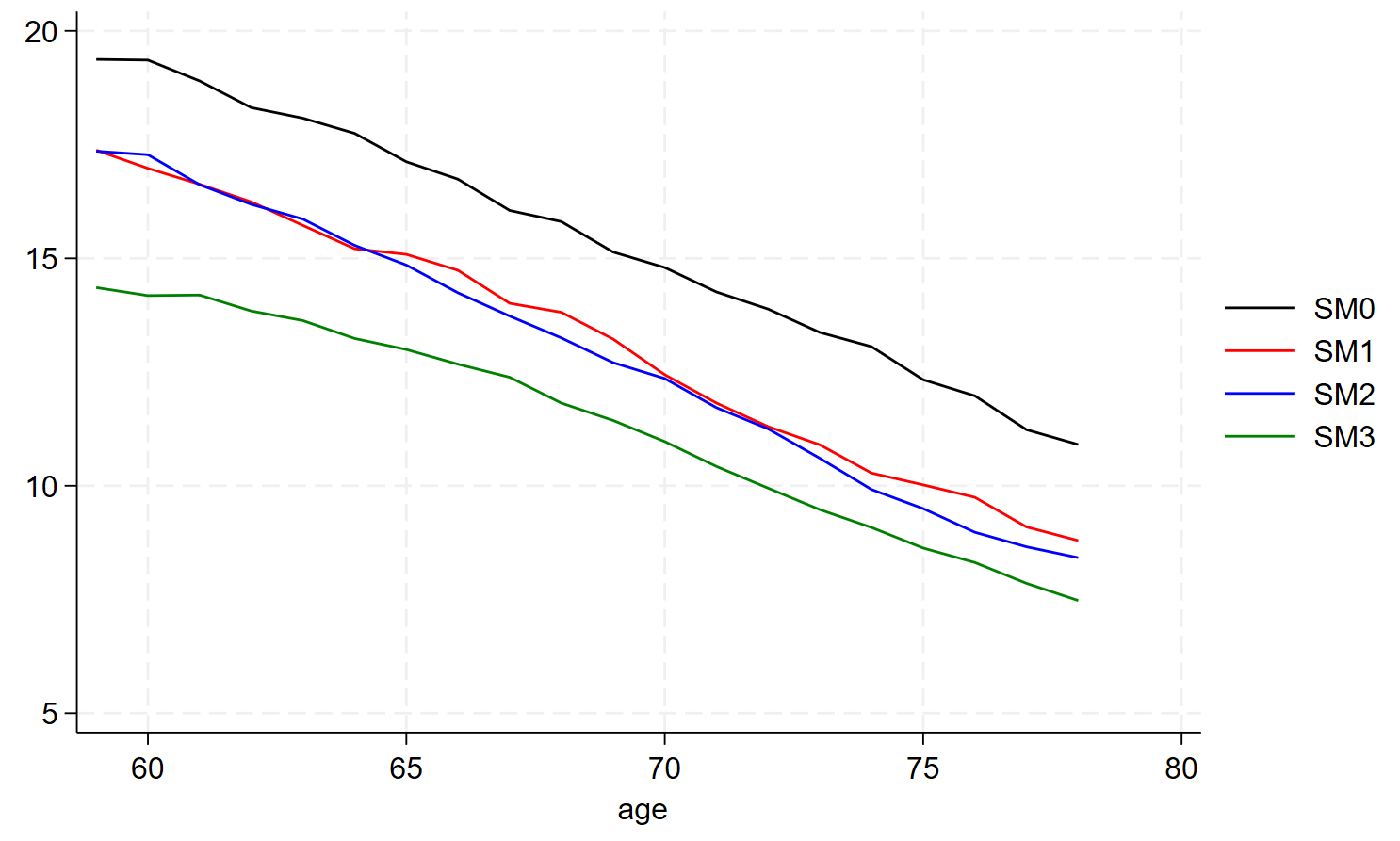


Remaing Life Expectancy

**Supp Figure 4.** Men. Colorectal cancer. Remaining Life Expentancy.

Cancer diagnosis between 59 and 78 years.
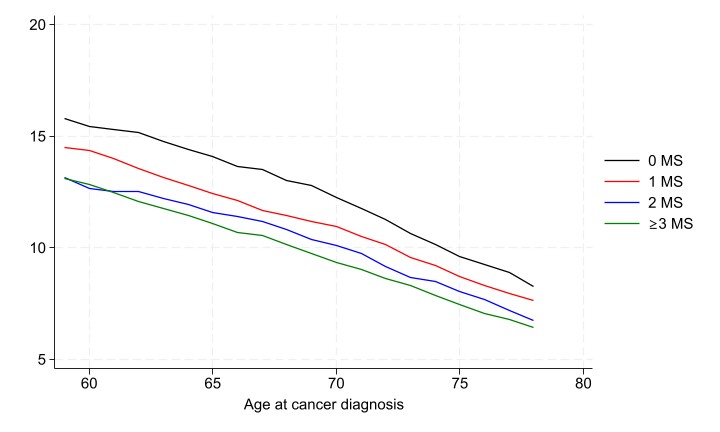


Remaing Life Expectancy

**Supp Figure 5.** Women. Liver cancer. Remaining Life Expentancy.

Cancer diagnosis between 59 and 78 years.


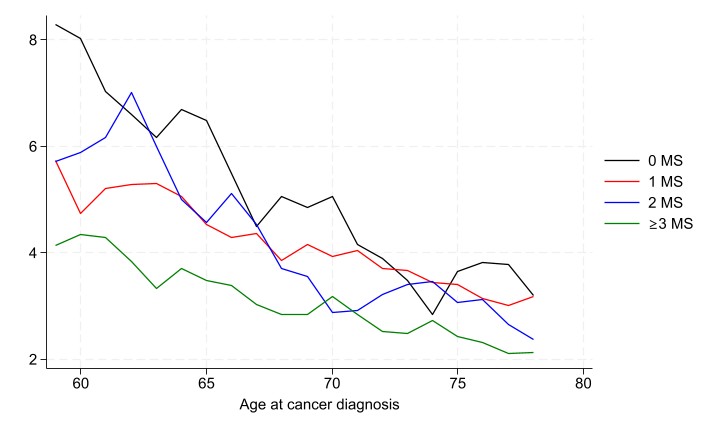


Remaing Life Expectancy

**Supp Figure 6.** Men. Liver cancer. Remaining Life Expentancy.

Cancer diagnosis between 59 and 78 years.
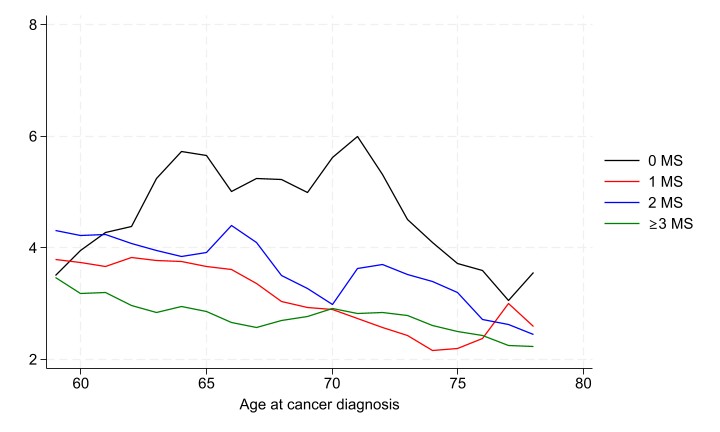


Remaing Life Expectancy

**Supp Figure 7.** Women. Pancreas cancer. Remaining Life Expentancy.

Cancer diagnosis between 59 and 78 years.


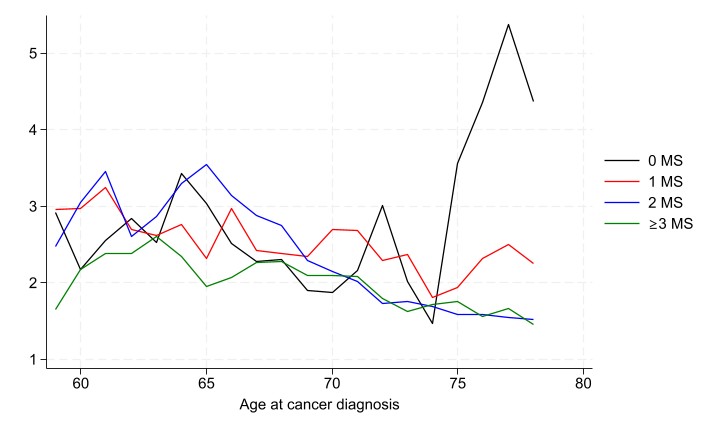


Remaing Life Expectancy

**Supp Figure 8.** Men. Pancreas cancer. Remaining Life Expentancy.

Cancer diagnosis between 59 and 78 years.
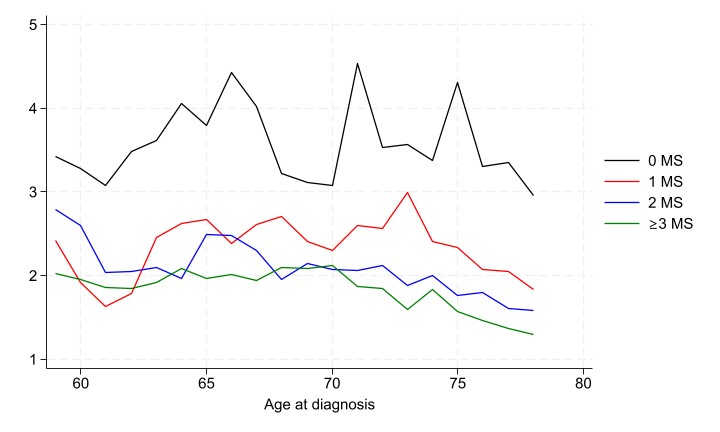


Remaing Life Expectancy

**Supp Figure 9.** Women. Breast cancer. Remaining Life Expentancy.

Cancer diagnosis between 59 and 78 years.


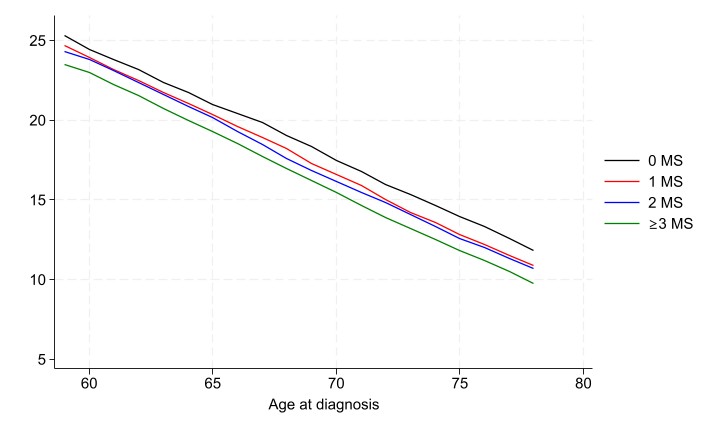


Remaing Life Expectancy

**Supp Figure 10.** Women. Endometrial cancer. Remaining Life Expentancy.

Cancer diagnosis between 59 and 78 years.
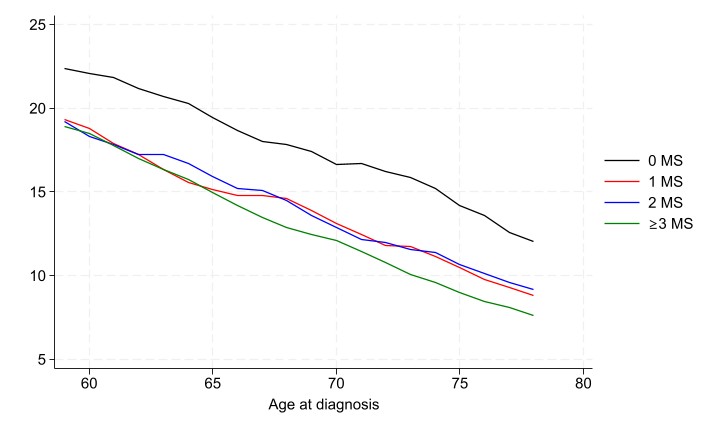


Remaing Life Expectancy

**Supp Figure 11.** Women. Bladder cancer. Remaining Life Expentancy.

Cancer diagnosis between 59 and 78 years.


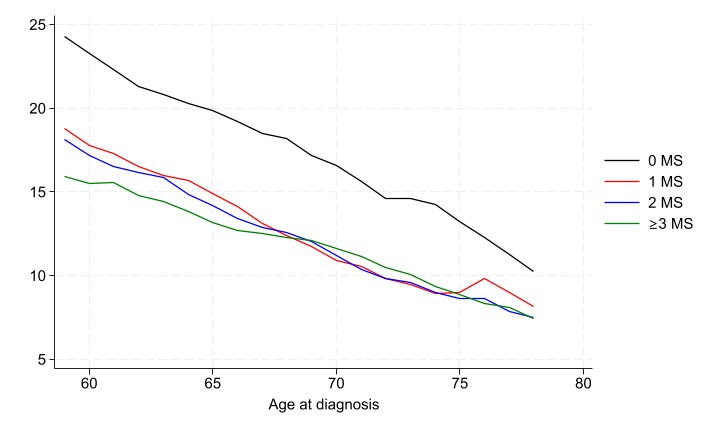


Remaing Life Expectancy

**Supp Figure 12.** Men. Bladder cancer. Remaining Life Expentancy.

Cancer diagnosis between 59 and 78 years.
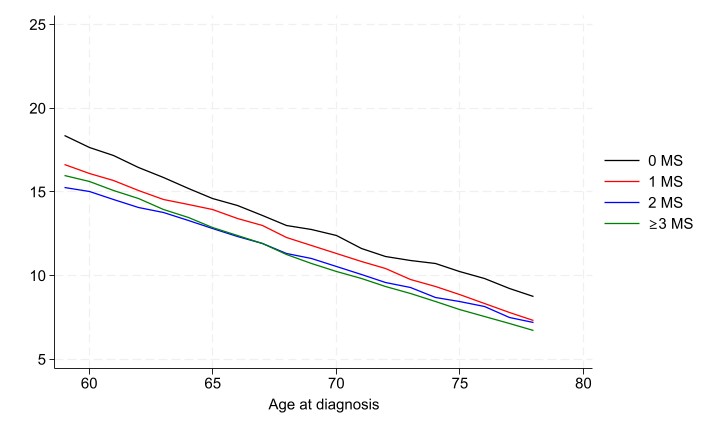


Remaing Life Expectancy

**Supp Figure 13.** Women. Kidney cancer. Remaining Life Expentancy.

Cancer diagnosis between 59 and 78 years.


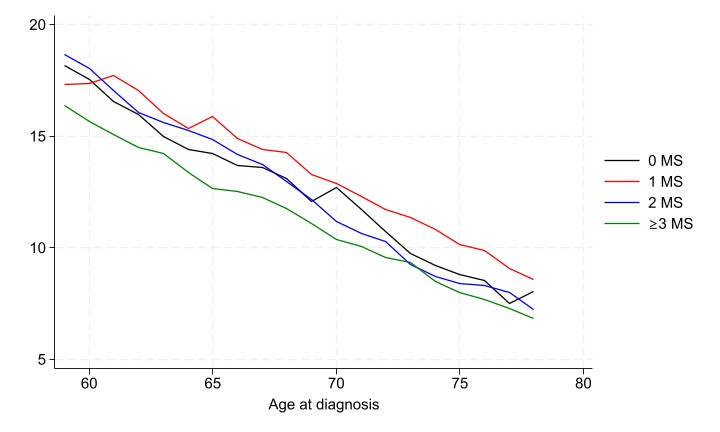


Remaing Life Expectancy

**Supp Figure 14.** Men. Kidney cancer. Remaining Life Expentancy.

Cancer diagnosis between 59 and 78 years.
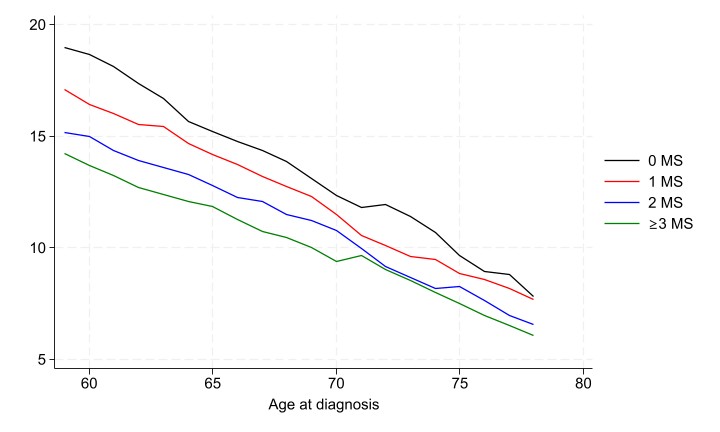


Remaing Life Expectancy

**Supp Figure 15.** Men. Prostate cancer. Remaining Life Expentancy.

Cancer diagnosis between 59 and 78 years.


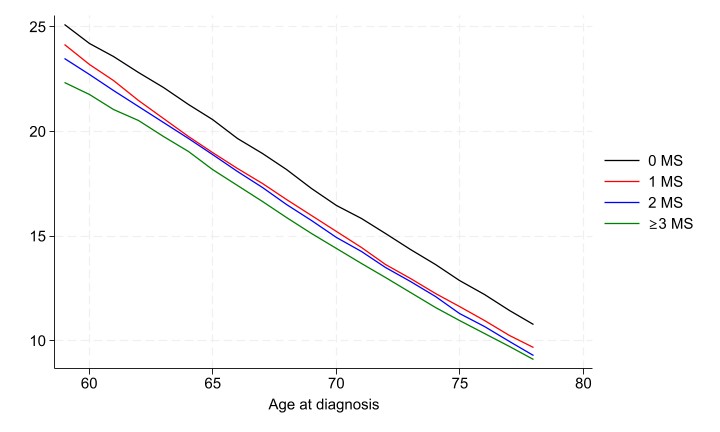


Remaing Life Expectancy

**Supp Figure 16.** Women. Hodgkin lymphoma. Remaining Life Expentancy.

Cancer diagnosis between 59 and 78 years.


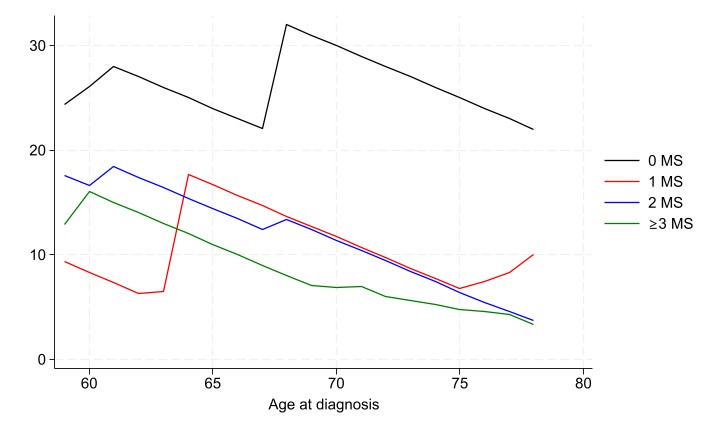


Remaing Life Expectancy

**Supp Figure 17.** Men. Hodgkin lymphoma. Remaining Life Expentancy.

Cancer diagnosis between 59 and 78 years.
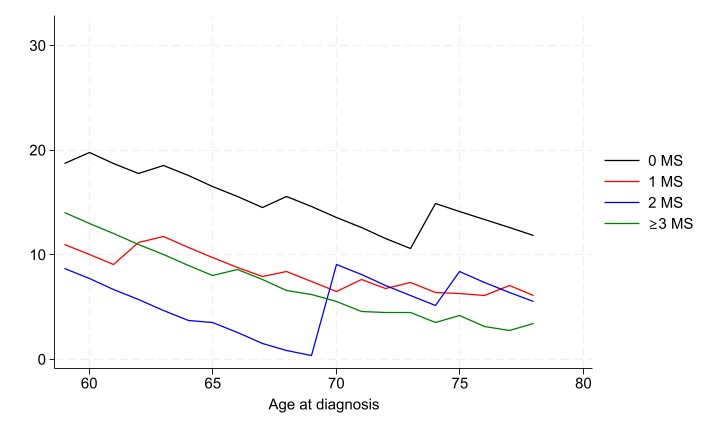


Remaing Life Expectancy

**Supp Figure 18.** Women. Non-Hodgkin lymphoma. Remaining Life Expentancy.

Cancer diagnosis between 59 and 78 years.


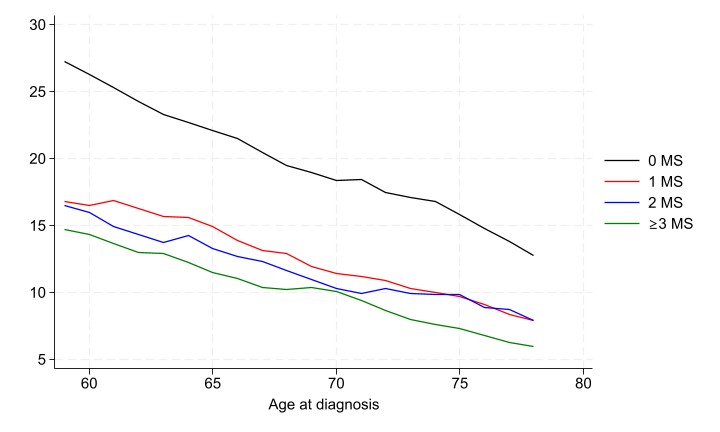


Remaing Life Expectancy

**Supp Figure 19.** Men. Non-Hodgkin lymphoma. Remaining Life Expentancy.

Cancer diagnosis between 59 and 78 years.
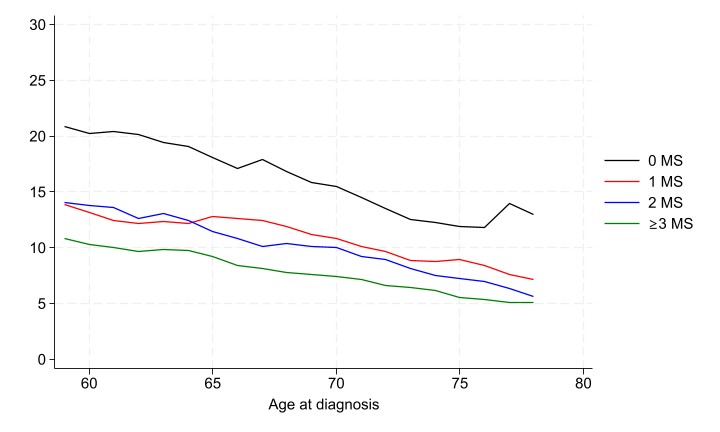


Remaing Life Expectancy

**Supp Figure 20.** Women. Leukemia. Remaining Life Expentancy.

Cancer diagnosis between 59 and 78 years.


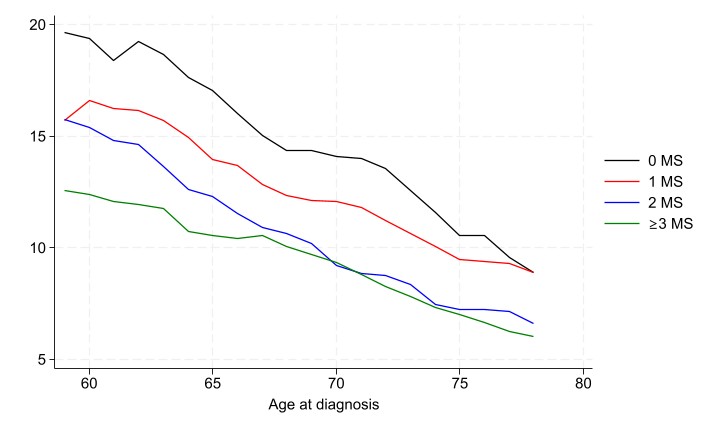


Remaing Life Expectancy

**Supp Figure 21.** Men. Leukemia. Remaining Life Expentancy.

Cancer diagnosis between 59 and 78 years.
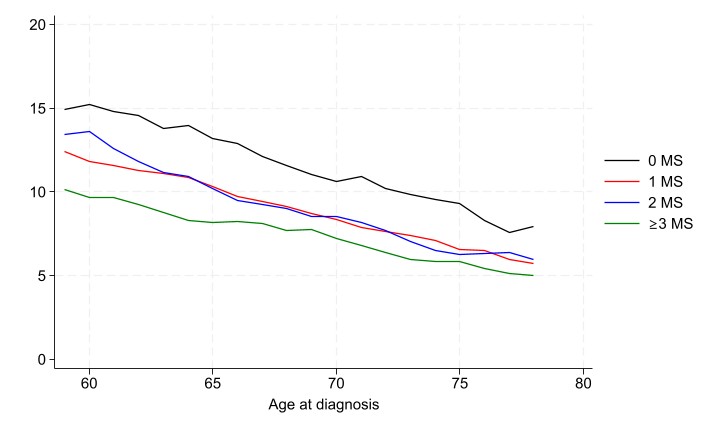


Remaing Life Expectancy

**Supp Figure 22.** Women. Lung cancer. Remaining Life Expentancy.

Cancer diagnosis between 59 and 78 years.


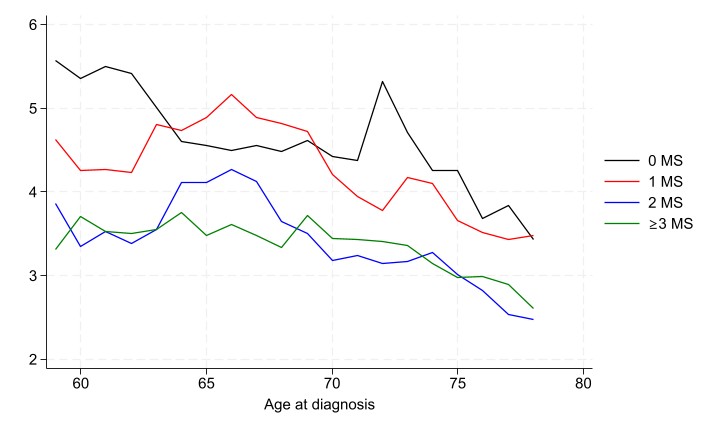


Remaing Life Expectancy

**Supp Figure 23.** Men. Lung cancer. Remaining Life Expentancy.

Cancer diagnosis between 59 and 78 years.
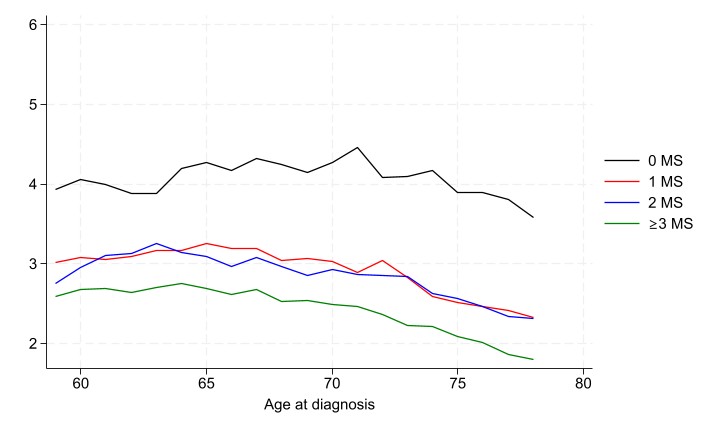


Remaing Life Expectancy

**Supp Figure 24.** Women. Thyroid cancer. Remaining Life Expentancy.

Cancer diagnosis between 59 and 78 years.


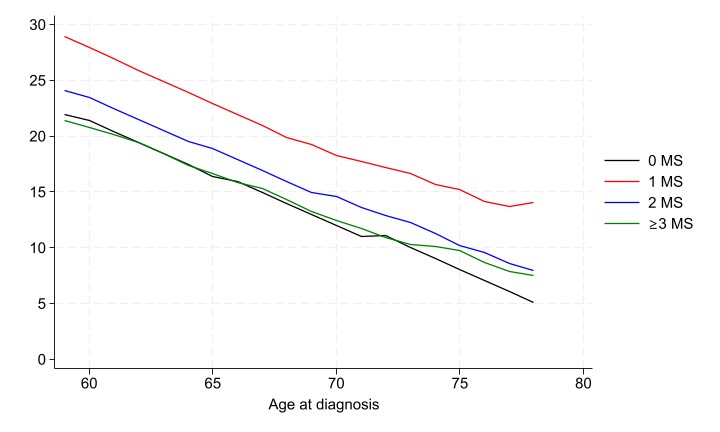


Remaing Life Expectancy

**Supp Figure 25.** Men. Thyroid cancer. Remaining Life Expentancy.

Cancer diagnosis between 59 and 78 years.


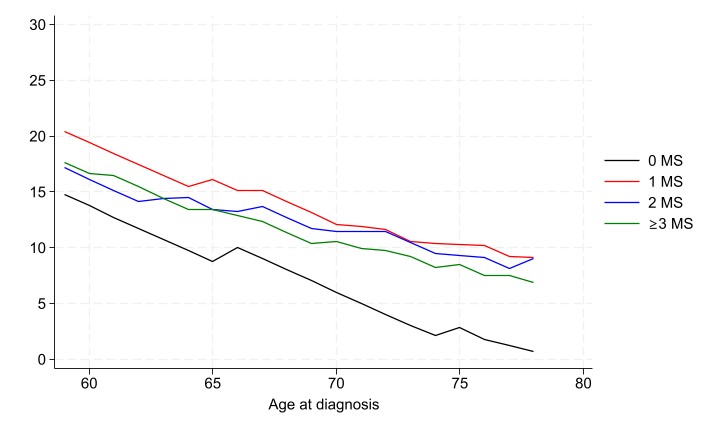


Remaing Life Expectancy

**Supp Figure 26.** WOMEN: 5 years survival rate by cancer type
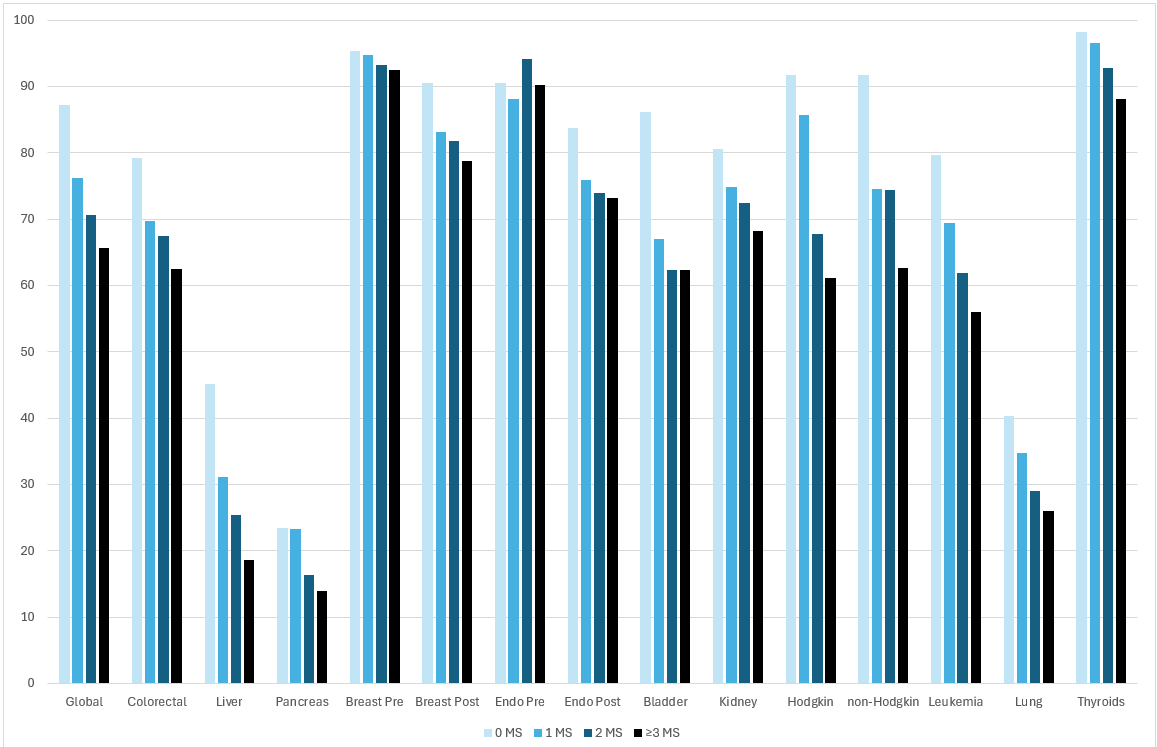


**Supp Figure 27.** MEN: 5 years survival rate by cancer type.


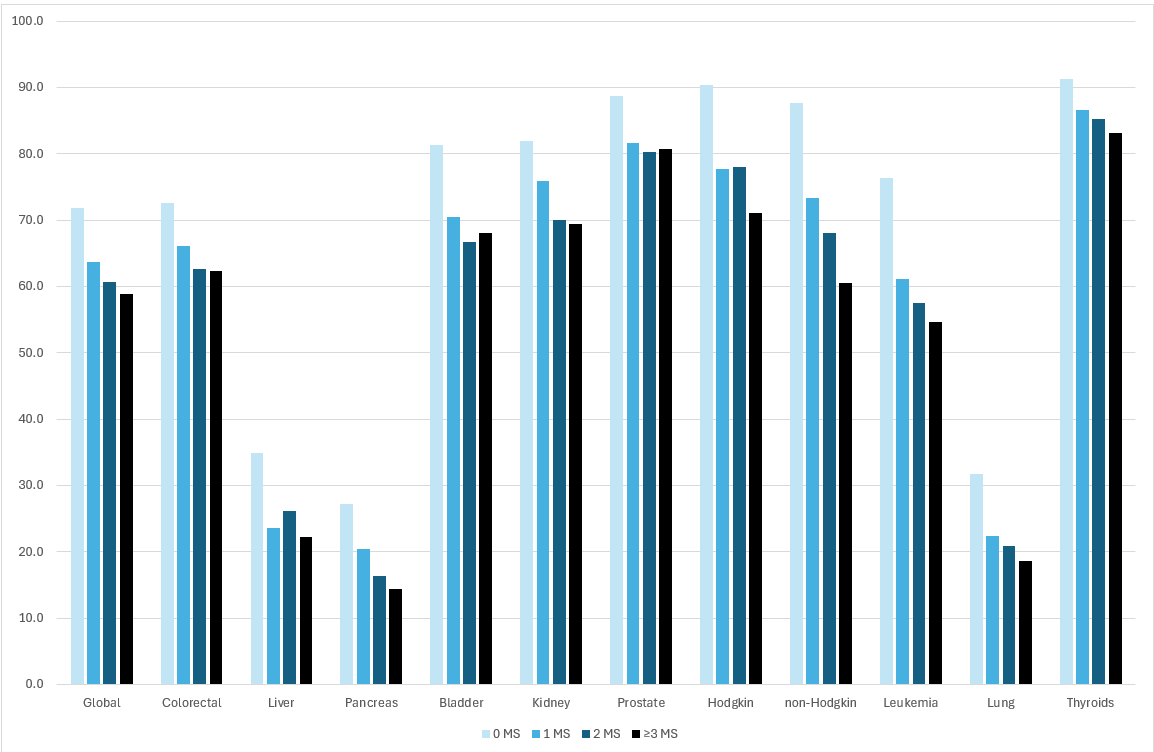


**Supp Figure 28.** Women with Colorectal Cancer. Kaplan Meier Curve by MS.
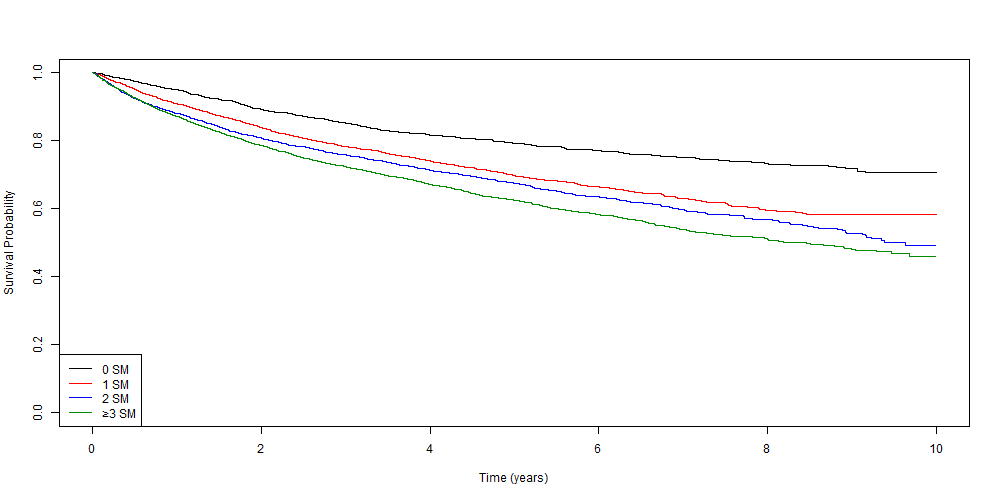


Log rank test <0.0001

**Supp Figure 29.** Men with Colorectal Cancer. Kaplan Meier Curve by MS.
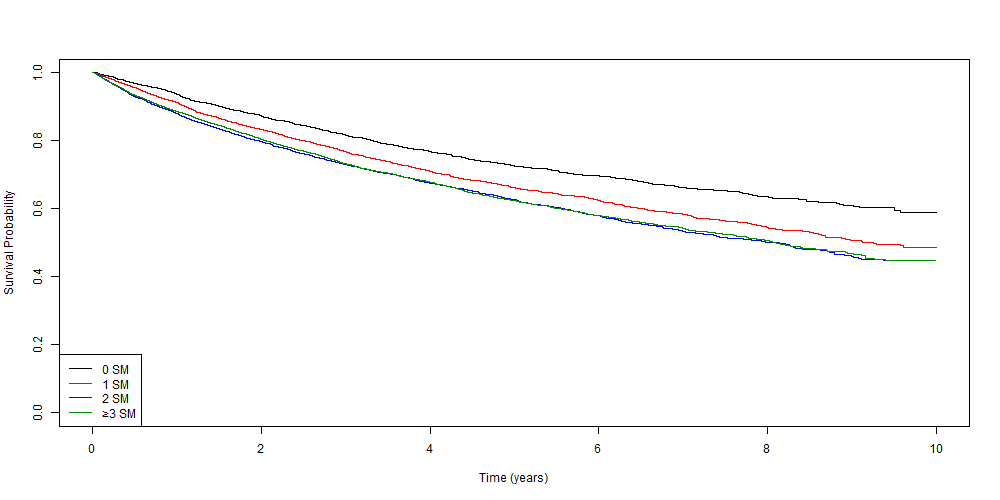


Log rank test <0.0001

**Supp Figure 30.** Women with Liver Cancer. Kaplan Meier Curve by MS.
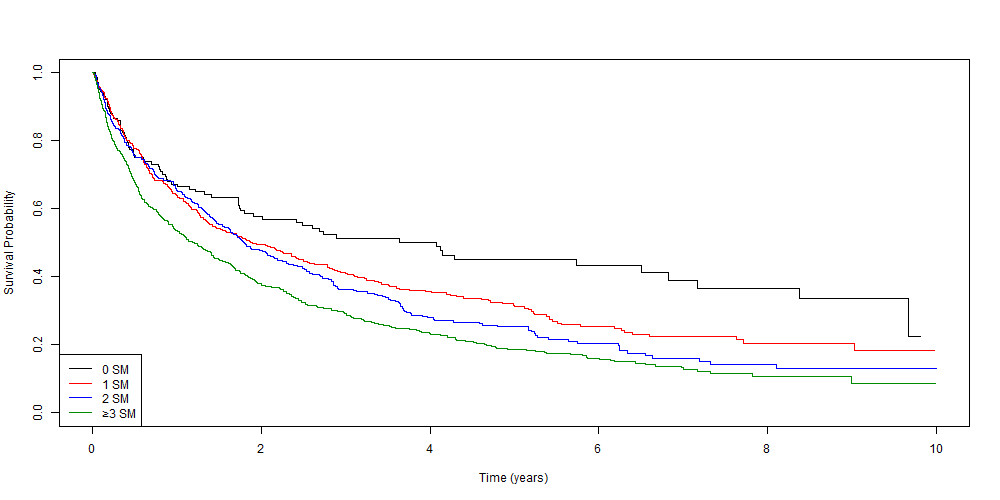


Log rank test <0.0001

**Supp Figure 31.** Men with Liver Cancer. Kaplan Meier Curve by MS.
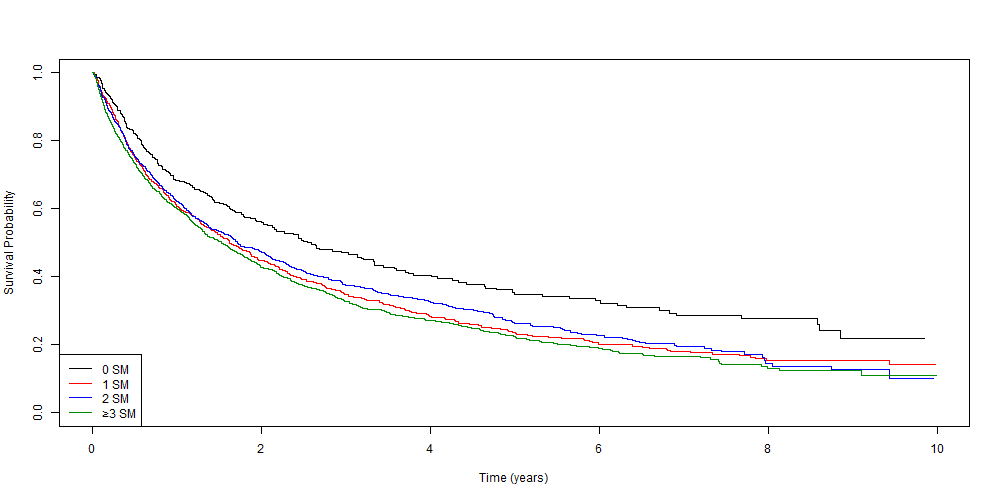


Log rank test <0.0001

**Supp Figure 32.** Women with Pancreas Cancer. Kaplan Meier Curve by MS.
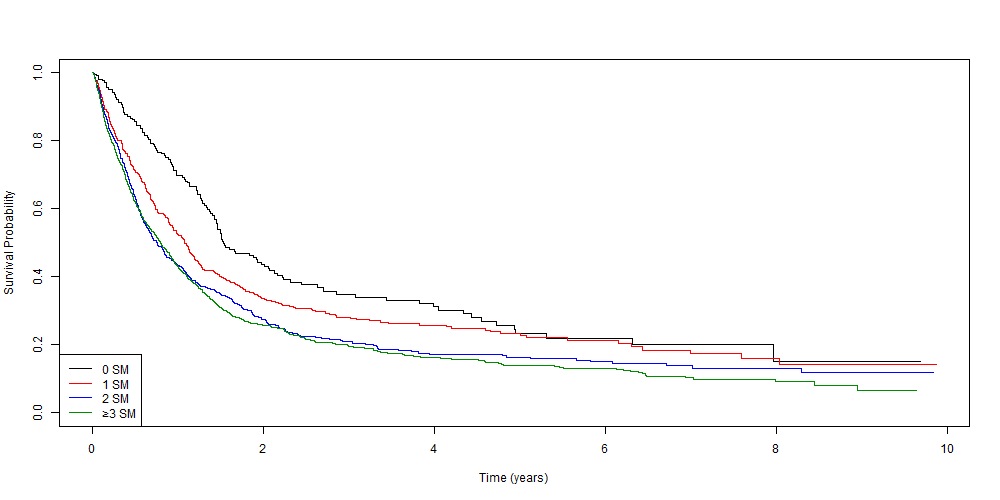


Log rank test <0.0001

**Supp Figure 33.** Men with Pancreas Cancer. Kaplan Meier Curve by MS.
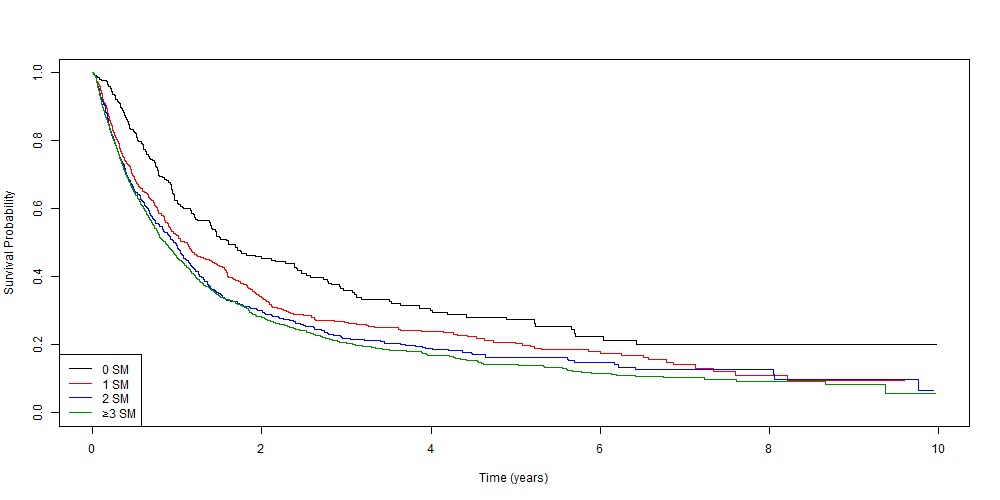


Log rank test <0.0001

**Supp Figure 34.** Women with Breast Cancer. Kaplan Meier Curve by MS
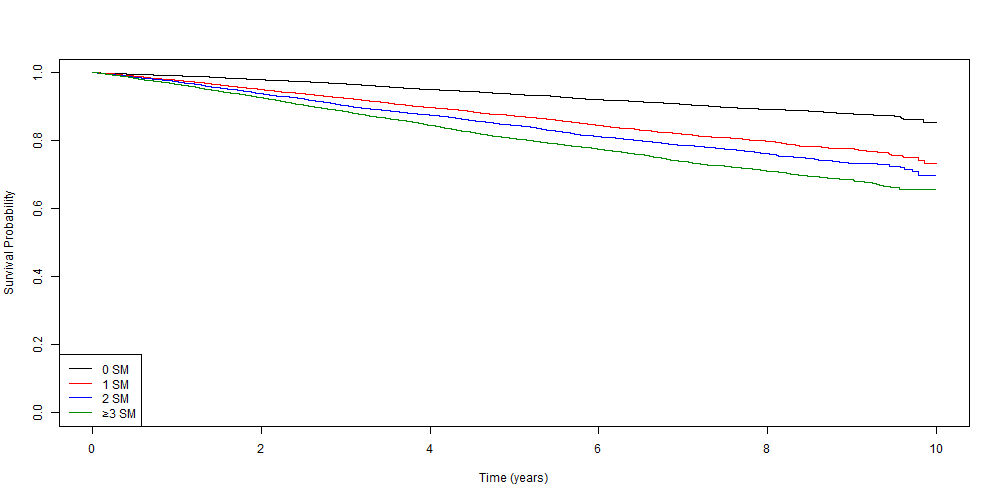


Log rank test <0.0001

**Supp Figure 35.** Women Pre-Menopausal with Breast Cancer. Kaplan Meier Curve by MS.
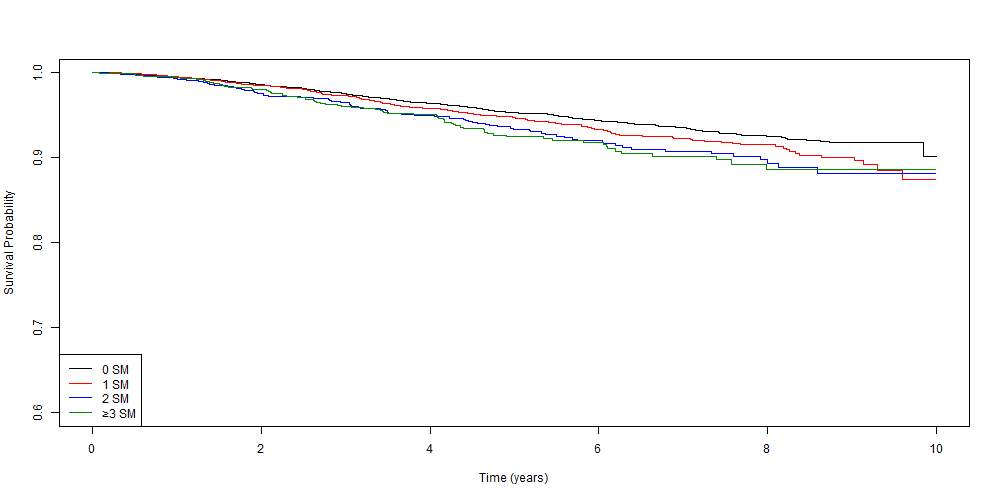


Log rank test = 0.0004

**Supp Figure 36.** Women Post-Menopausal with Breast Cancer. Kaplan Meier Curve by MS.
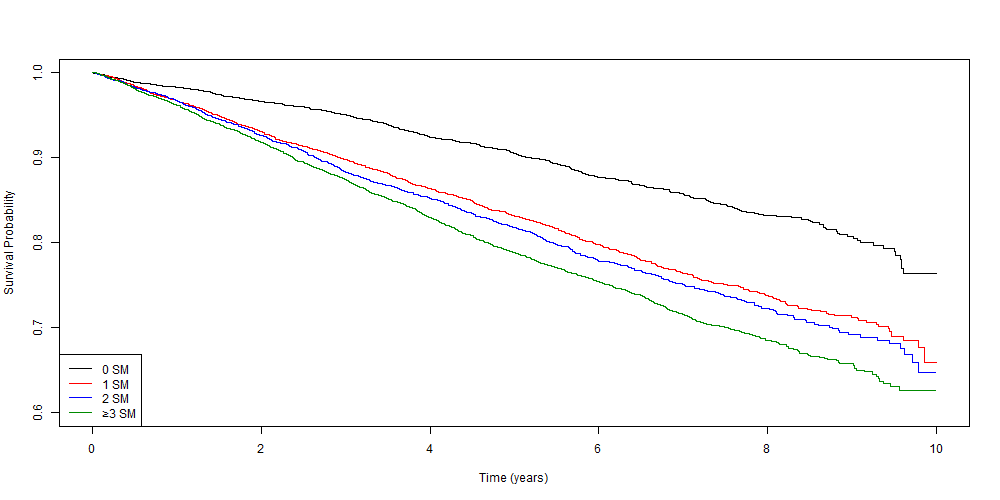


Log rank test <0.0001

**Supp Figure 37.** Women with Endometrial Cancer. Kaplan Meier Curve by MS
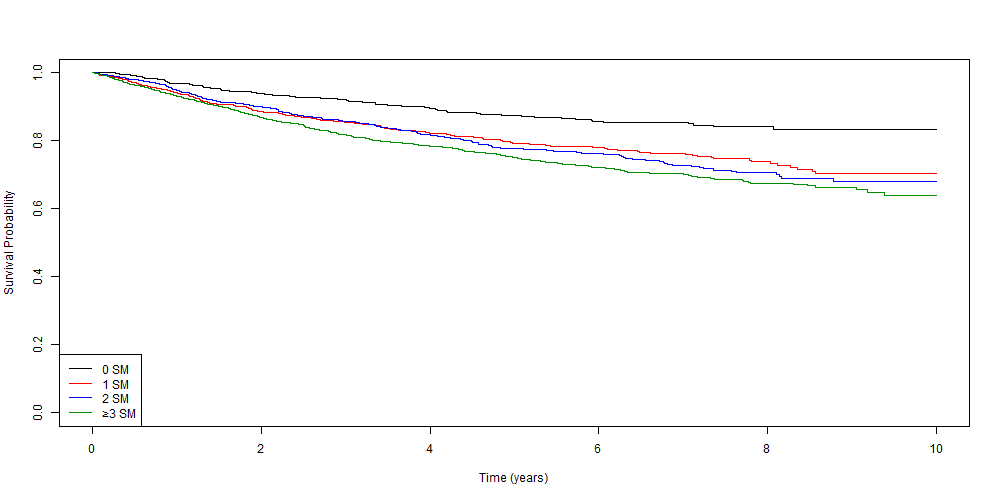


Log rank test <0.0001

**Supp Figure 38.** Women Pre-Menopausal with Endometrial Cancer. Kaplan Meier Curve by MS.
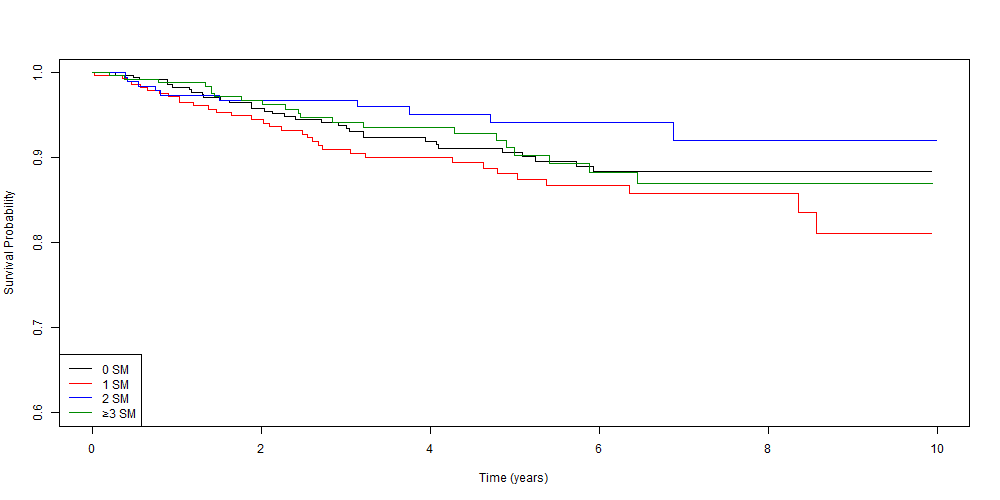


Log rank test <0.0001

**Supp Figure 39.** Women Post-Menopausal with Endometrial Cancer. Kaplan Meier Curve by MS.
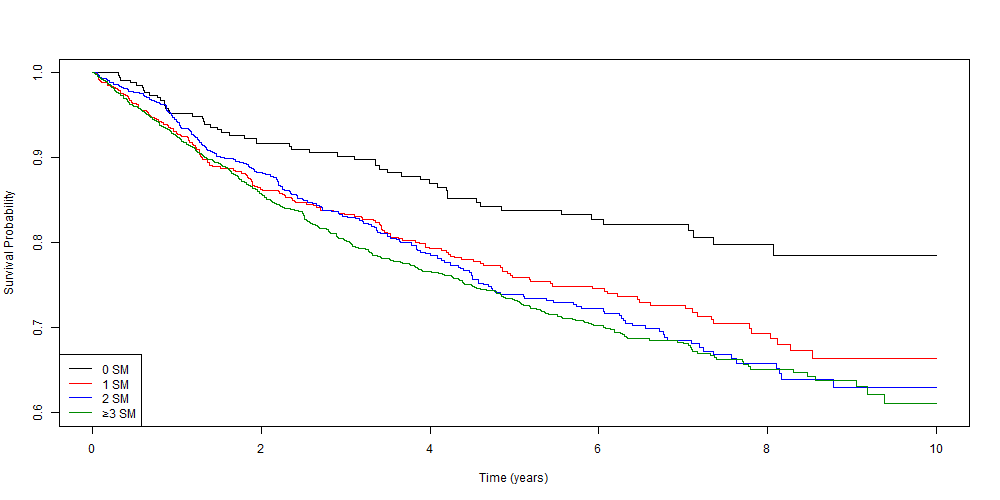


Log rank test = 0.0001

**Supp Figure 40.** Women with Bladder Cancer. Kaplan Meier Curve by MS.
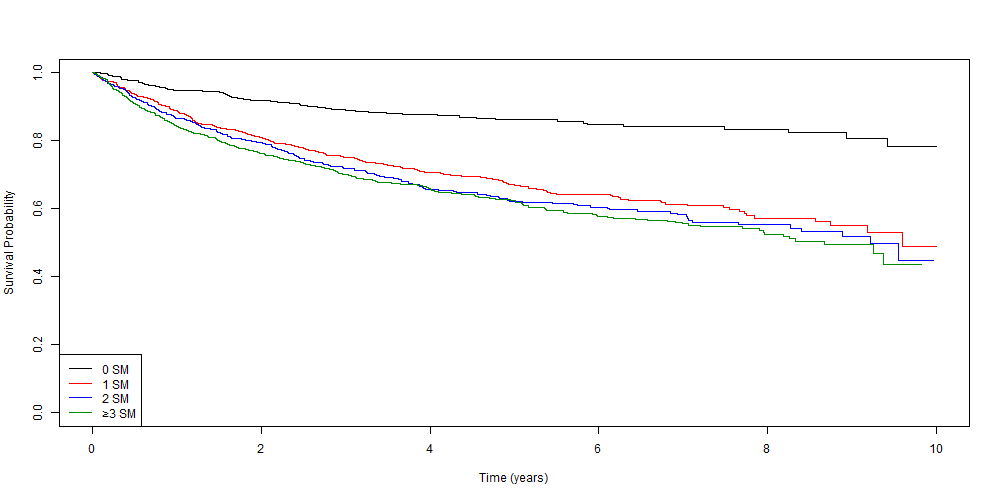


Log rank test <0.0001

**Supp Figure 41.** Men with Bladder Cancer. Kaplan Meier Curve by MS.
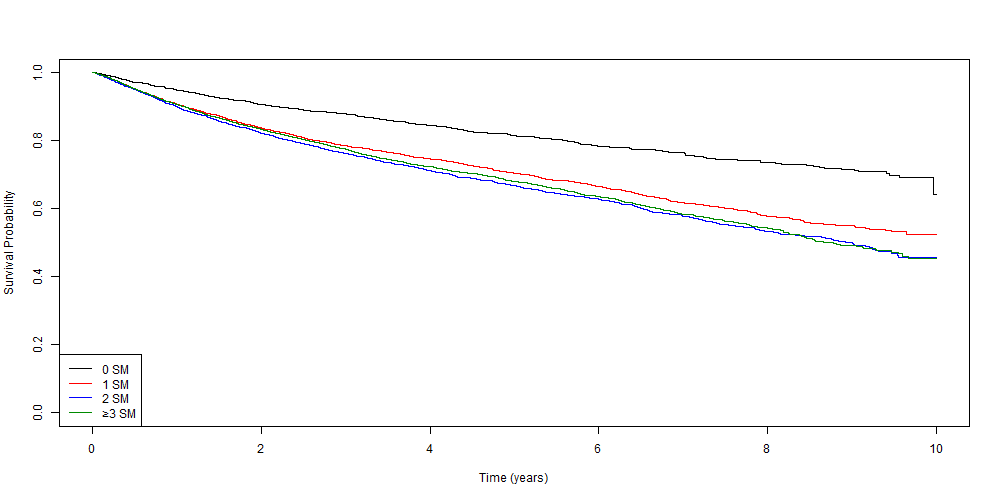


Log rank test <0.0001

**Supp Figure 42.** Women with Kidney Cancer. Kaplan Meier Curve by MS.
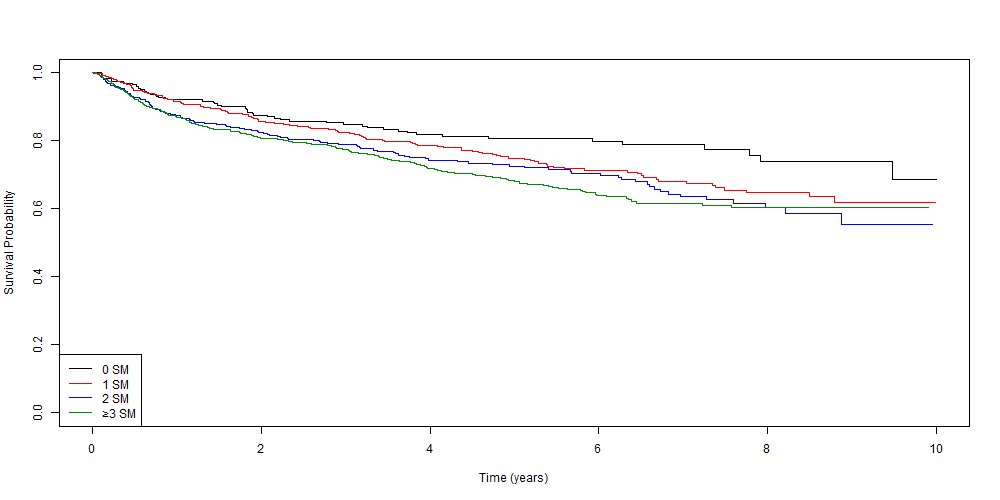


Log rank test = 0.0005

**Supp Figure 43.** Men with Kidney Cancer. Kaplan Meier Curve by MS.
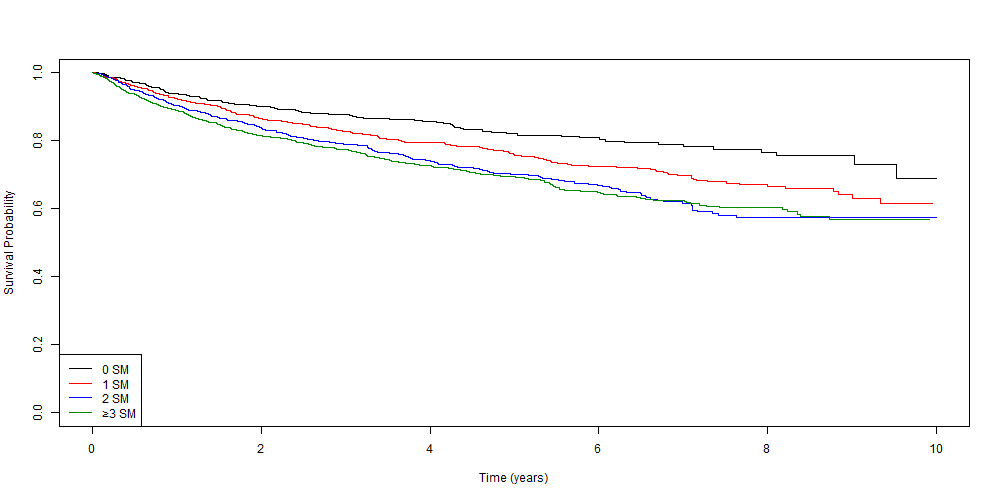


Log rank test <0.0001

**Supp Figure 44.** Men with Prostate Cancer. Kaplan Meier Curve by MS.
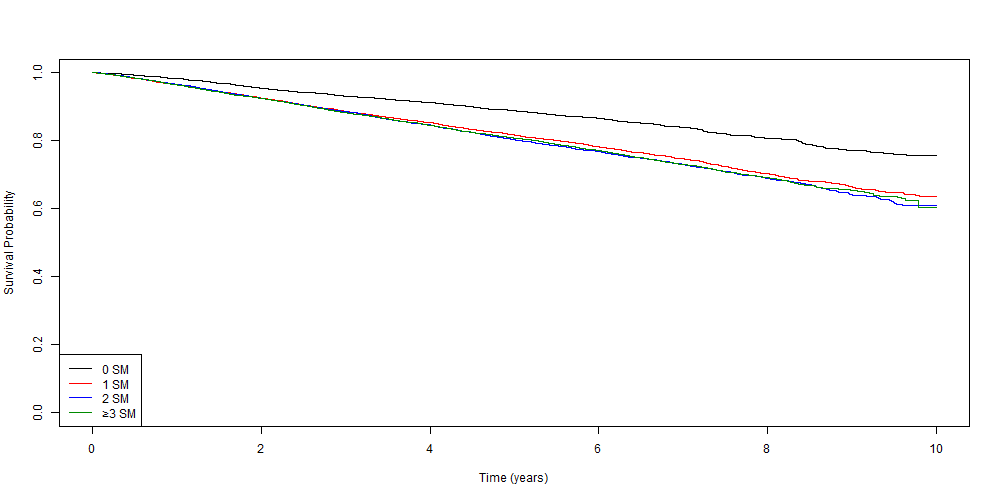


Log rank test <0.0001

**Supp Figure 45.** Women with Hodgkin lymphoma Kaplan Meier Curve by MS.
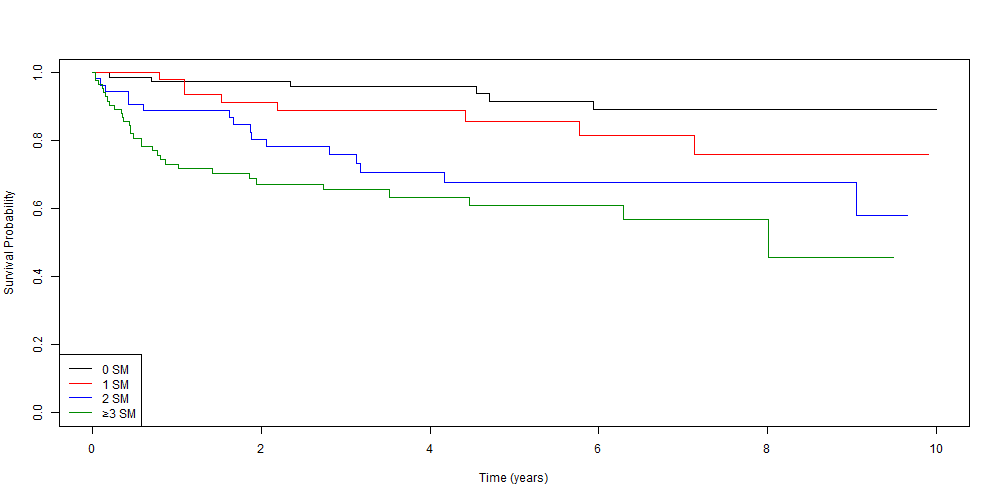


Log rank test <0.0001

**Supp Figure 46.** Men with Hodgkin lymphoma. Kaplan Meier Curve by MS.
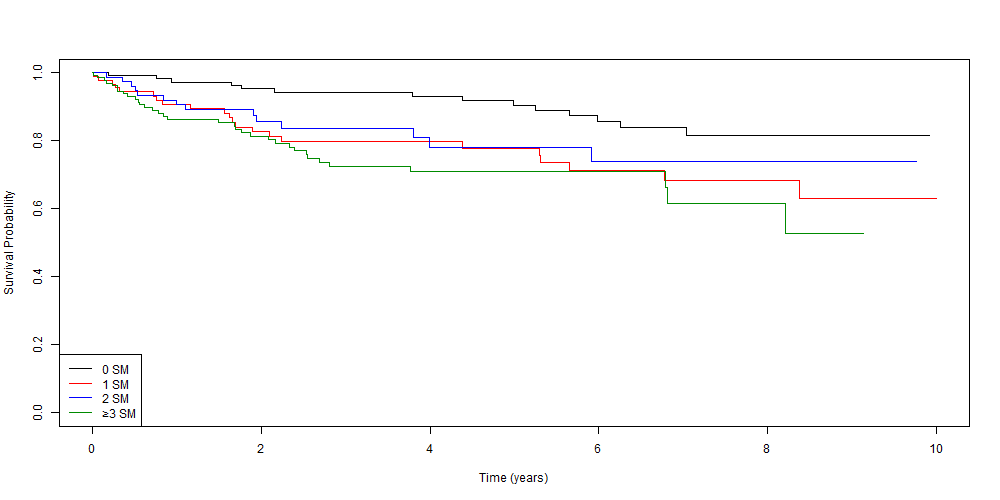


Log rank test = 0.003

**Supp Figure 47.** Women with Non-Hodgkin lymphoma. Kaplan Meier Curve by MS.
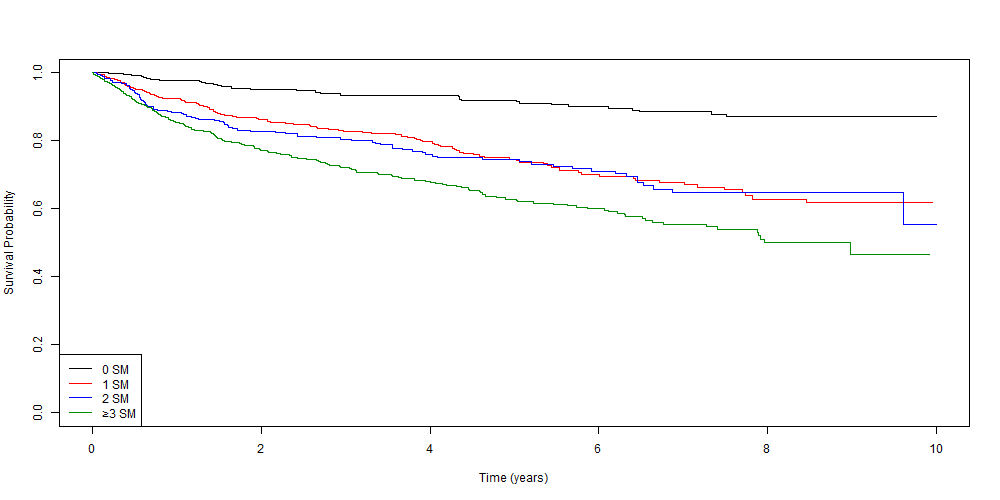


Log rank test <0.0001

**Supp Figure 48.** Men with Non-Hodgkin lymphoma. Kaplan Meier Curve by MS.
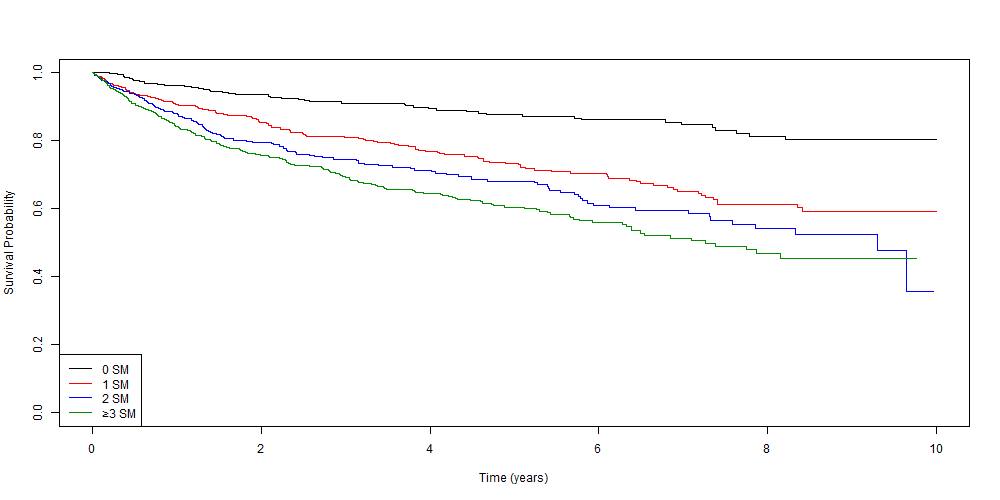


Log rank test <0.0001

**Supp Figure 49.** Women with Leukemia. Kaplan Meier Curve by MS.
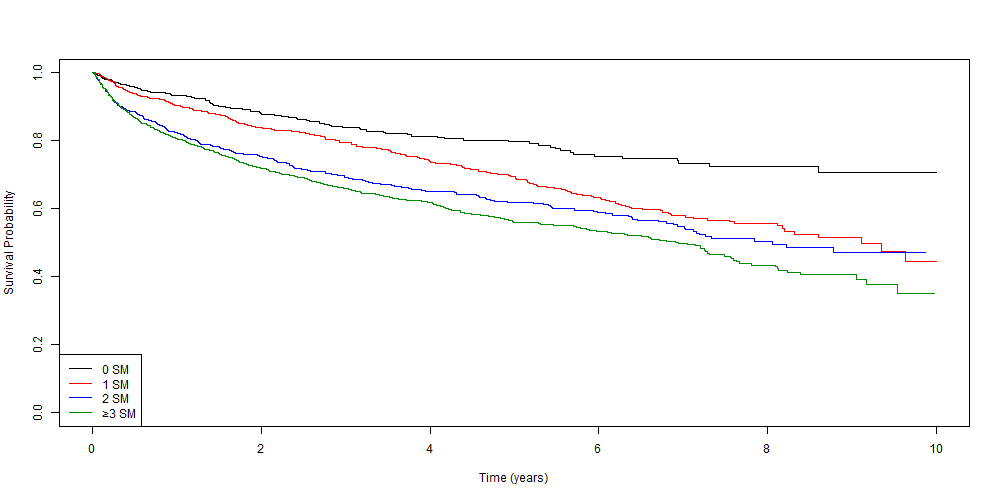


Log rank test <0.0001

**Supp Figure 50.** Men with Leukemia. Kaplan Meier Curve by MS.
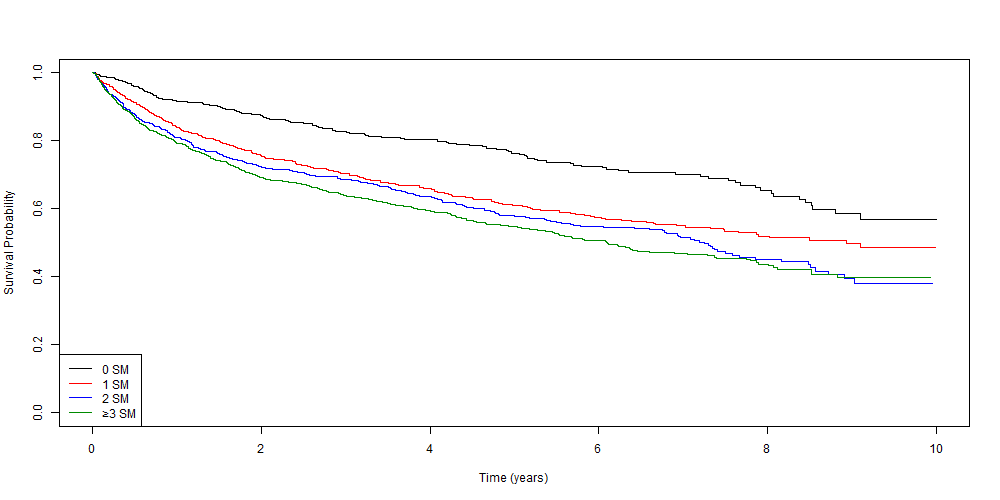


Log rank test <0.0001

**Supp Figure 51.** Women with Lung Cancer. Kaplan Meier Curve by MS.
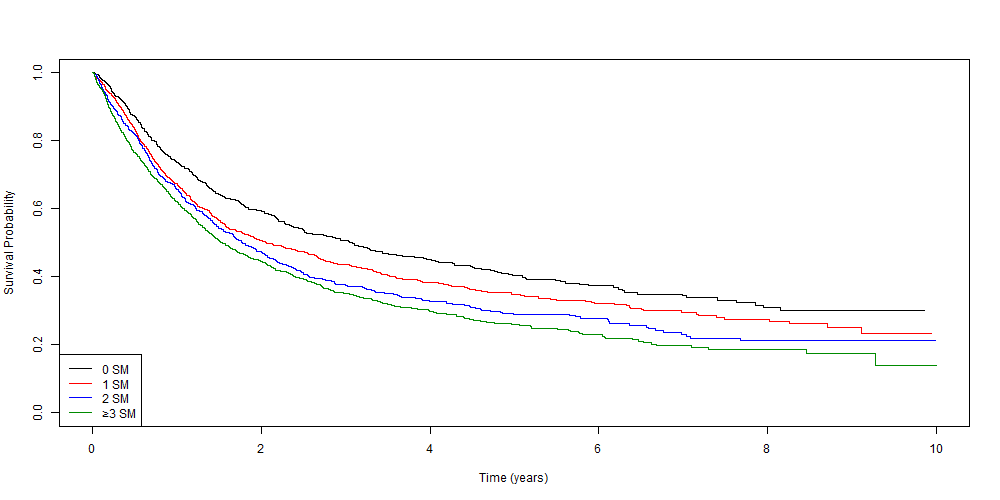


Log rank test <0.0001

**Supp Figure 52.** Men with Lung Cancer. Kaplan Meier Curve by MS.
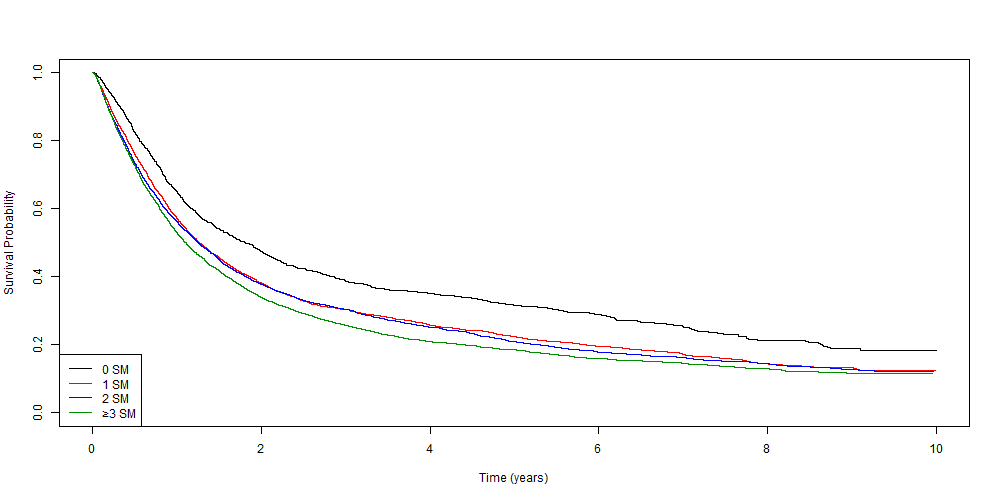


Log rank test <0.0001

**Supp Figure 53.** Women with Thyroid Cancer. Kaplan Meier Curve by MS.
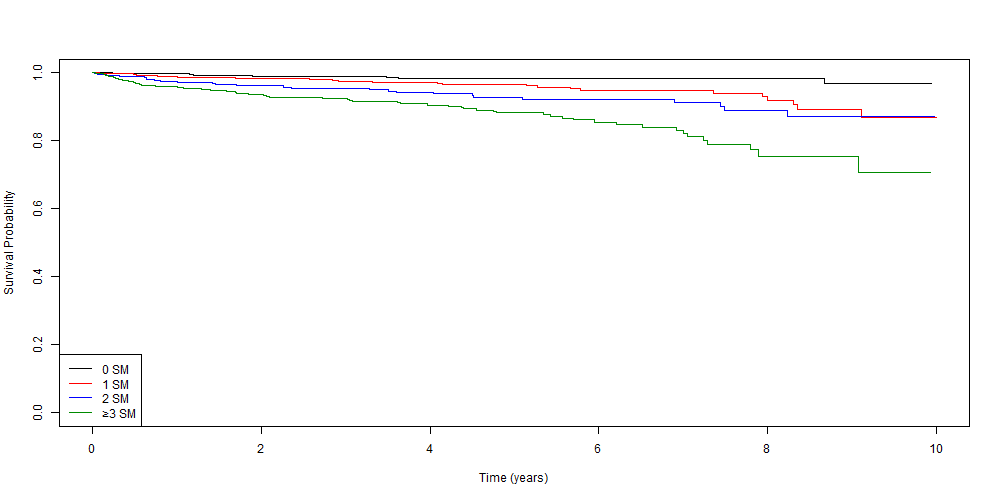


Log rank test <0.0001

**Supp Figure 54.** Men with Thyroid Cancer. Kaplan Meier Curve by MS.
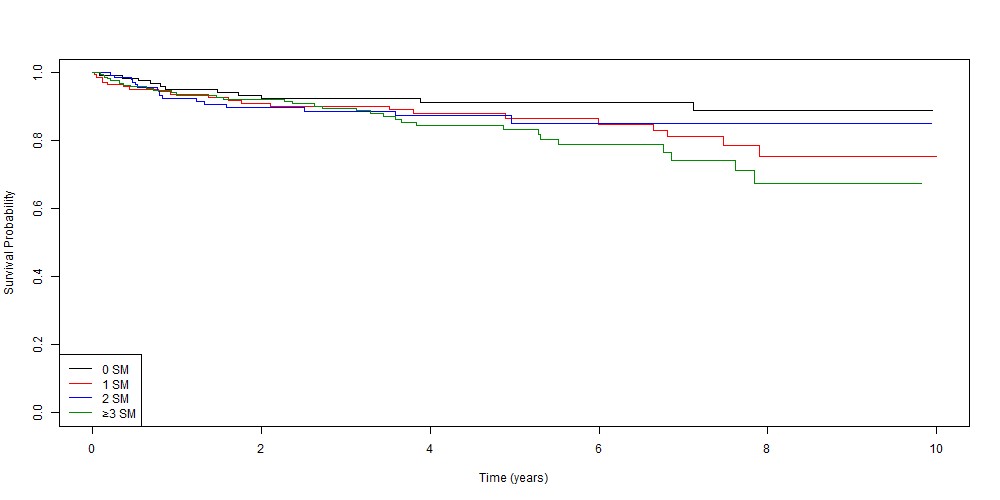


Log rank test = 0.100
